# A Novel Mechanism of Endoplasmic Reticulum Stress‐ and c‐Myc‐Degradation‐Mediated Therapeutic Benefits of Antineurokinin‐1 Receptor Drugs in Colorectal Cancer

**DOI:** 10.1002/advs.202101936

**Published:** 2021-10-03

**Authors:** Yue Shi, Xi Wang, Yueming Meng, Junjie Ma, Qiyu Zhang, Gang Shao, Lingfei Wang, Xurui Cheng, Xiangyu Hong, Yong Wang, Zhibin Yan, Yihai Cao, Jian Kang, Caiyun Fu

**Affiliations:** ^1^ Zhejiang Provincial Key Laboratory of Silkworm Bioreactor and Biomedicine College of Life Sciences and Medicine Zhejiang Sci‐Tech University Hangzhou 310018 China; ^2^ Department of Oncology No. 903 Hospital of PLA Joint Logistic Support Force Xi Hu Affiliated Hospital of Hangzhou Medical College Hangzhou 310013 China; ^3^ Department of Microbiology Tumor and Cell Biology Karolinska Institute Stockholm 171 77 Sweden; ^4^ Oncogenic Signalling and Growth Control Program Peter MacCallum Cancer Centre 305 Grattan Street Melbourne Victoria 3000 Australia; ^5^ Sir Peter MacCallum Department of Oncology University of Melbourne Melbourne Victoria 3000 Australia

**Keywords:** chemotherapy resistance, ER stress, human colorectal cancer, MAPK signal pathway, neurokinin‐1 receptor

## Abstract

The neurokinin‐1 receptor (NK‐1R) antagonists are approved as treatment for chemotherapy‐associated nausea and vomiting in cancer patients. The emerging role of the substance P‐NK‐1R system in oncogenesis raises the possibility of repurposing well‐tolerated NK‐1R antagonists for cancer treatment. This study reports that human colorectal cancer (CRC) patients with high NK‐1R expression have poor survival, and NK‐1R antagonists SR140333 and aprepitant induce apoptotic cell death in CRC cells and inhibit CRC xenograft growth. This cytotoxicity induced by treatment with NK‐1R antagonists is mediated by induction of endoplasmic reticulum (ER) stress. ER stress triggers calcium release, resulting in the suppression of prosurvival extracellular signal‐regulated kinase (ERK)‐c‐Myc signaling. Along with ER calcium release, one ER stress pathway mediated by protein kinase RNA‐like ER kinase (PERK) is specifically activated, leading to increased expression of proapoptotic C/EBP‐homologous protein (CHOP). Moreover, NK‐1R antagonists enhance the efficacy of chemotherapy by increasing the sensitivity and overcoming resistance to 5‐fluorouracil in CRC cells through the induction of sustained ER stress and the consequent suppression of ERK‐c‐Myc signaling both in vitro and in vivo. Collectively, the findings provide novel mechanistic insights into the efficacy of NK‐1R antagonists either as a single agent or in combination with chemotherapy for cancer treatment.

## Graphical abstract

The neurokinin‐1 receptor antagonists are repurposed as a new treatment option for patients with colorectal cancer via activation of endoplasmic reticulum stress to induce apoptosis.

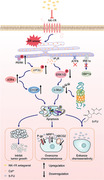

## 1 Introduction

Colorectal cancer (CRC) is the third most common cancer diagnosed in both women and men, with high mortality rates.^[^ ^1^ ^]^ In particular, the incidence of CRC in young adults aged less than 50 years has been increasing by 2.2% annually since 1995.^[^ ^2^ ^]^ With the current standard therapies including surgery, chemotherapy, and radiotherapy, the 5‐year relative survival rate of patients with CRC is 65%; however, it declines to 12% in patients with stage IV CRC.^[^ ^3^ ^]^ Thus, understanding the pathogenesis of CRC and development of new therapies are an unmet clinical need for CRC patients.

Substance P (SP) is the first identified member of the tachykinin family and is widely distributed in the central and peripheral nervous systems. SP regulates a variety of physiological and pathological processes such as pain, gastric motility, inflammation, immunomodulation, emesis, and depression.^[^ ^4^ ^]^ The biological responses to SP are mediated by the neurokinin receptors (NKRs), members of the G‐protein‐coupled receptor family, including NK‐1R, NK‐2R, and NK‐3R, of which NK‐1R confers the highest affinity to SP.^[^ ^4^ ^]^ NK‐1R antagonists have been used clinically for the treatment of nausea and vomiting caused by cancer chemotherapy.^[^ ^5^ ^]^

Accumulating evidence supports the oncogenic role of the SP/NK‐1R complex in the development of CRC.^[^ ^6^ ^]^ NK‐1R was preferentially upregulated in the colonic epithelial cells of patients with ulcerative colitis who later developed colonic carcinoma.^[^ ^7^ ^]^ An analysis of genetic variants of tachykinin precursor 1 (*TAC1*), tachykinin receptor 1 (*TACR1*), and tachykinin receptor 2 (*TACR2*) genes in patients with CRC and cancer‐free controls suggested that these genes may contribute to the development of CRC.^[^ ^8^ ^]^ Furthermore, a higher SP and NK‐1R expression in CRC tumor tissues than in adjacent normal tissues has been reported.^[^ ^9^ ^]^ Moreover, NK‐1R antagonists inhibit tumor growth and induce apoptosis in human colon cancer cells that express high levels of NK‐1R.^[^ ^10^ ^]^ However, the molecular mechanisms underlying the efficacy of NK‐1R antagonists in cancer cells have not been well characterized. Although a recent study reported that the cytotoxicity of NK‐1R antagonist aprepitant in human colon cancer cells was mediated by inhibition of the canonical Wnt pathway, the efficacy of aprepitant was independent of the basal Wnt activity,^[^ ^10a^ ^]^ suggesting that other pathways could be involved. Therefore, we performed an in‐depth mechanistic characterization to understand the sensitivity of human colon cancer cells to NK‐1R antagonists.

We uncovered that apoptosis induced by treatment with NK‐1R antagonists in CRC cells is mediated by the induction of endoplasmic reticulum (ER) stress via ER calcium release, which results in the inactivation of extracellular signal‐regulated kinase (ERK) signaling and leading to avian myelocytomatosis viral oncogene homolog (c‐Myc) protein degradation, and specifically activation of the protein kinase RNA‐like ER kinase (PERK)‐eukaryotic inition factor 2 (eIF2)*α*‐ activating transcription factor 4 (ATF4)‐C/EBP‐homologous protein (CHOP) ER stress signaling pathway. This novel mechanism of ER stress‐mediated cytotoxicity contributed to the improved cellular response to chemotherapy by NK‐1R antagonists in both chemosensitive and chemoresistant CRC cells. These findings provide a conceptual framework to expand our understanding of the NK‐1R‐mediated G‐protein‐coupled receptor (GPCR) signaling network and ER stress in cancer cells. Moreover, this research identified the potential predictive biomarkers and novel vulnerabilities for therapeutic targeting, which will facilitate the clinical development of NK‐1R antagonists for newly diagnosed and relapsed human CRC.

## 2 Results

### 2.1 Association between Positive NK‐1R Expression and Poor Survival in CRC Patients and Induction of Apoptosis by Targeting NK‐1R in Human CRC Cells

**Figure 1:**
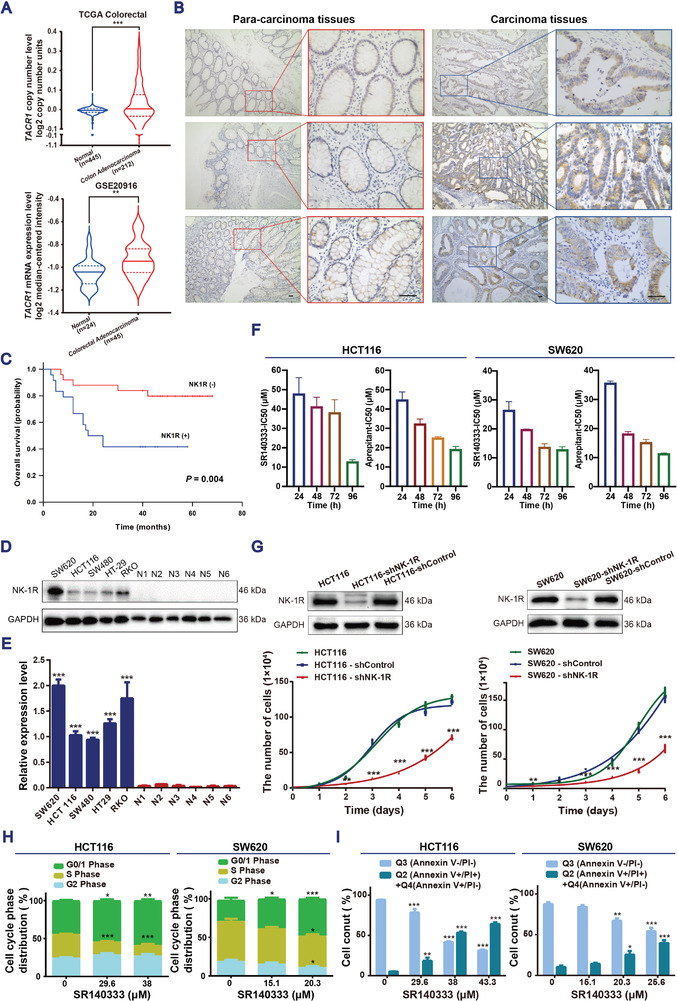
NK‐1R expression is associated with poor survival in CRC patients and targeting NK‐1R inhibits cell proliferation in human colon cancer cells. A) Oncomine data show *TACR1* copy number and mRNA expression levels in colon adenocarcinoma and colorectal adenocarcinoma cancer tissues, respectively. **P < 0.01, and ***P < 0.001 by student's *t*‐test. B) Representative images of paracarcinoma and carcinoma tissues of patients with colon cancer stained with NK‐1R. Scale bar, 50 µm. C) Kaplan–Meier survival analysis of 50 colon cancer patients stratified by NK‐1R expression level. *P* = 0.004 by log‐rank (Mantel–Cox) test. D) Western blotting of NK‐1R expression in human colon cancer cell lines and human normal colon tissues (abbreviation as N). Glyceraldehyde‐3‐phosphate dehydrogenase (GAPDH) was used as a loading control. Images are representative of three independent experiments. E) Quantification of total NK‐1R expression levels by densitometry and normalized with GAPDH in each sample. Data are expressed as mean ± SEM, *n* = 3, ***P < 0.001, compared with the normal controls by student's *t*‐test. F) The IC_50_ values of SR140333 or Aprepitant measured by 3‐(4,5‐dimethylthiazol‐2‐yl)‐2,5‐diphenyltetrazolium bromide (MTT) assay. Data are expressed as mean ± SEM, *n* = 3. G) Cells were transfected with either shRNA against NK‐1R or control shRNA. The cell proliferation curve measured by trypan blue exclusion assay and Western blotting showing NK‐1R expression. Data presented as mean ± SEM, *n* = 4, *P*‐values are calculated using one‐way ANOVA with Dunnett correction, *P < 0.05, **P < 0.01, ***P < 0.001. H) Cell cycle analysis by PI‐binding assay and I) annexin V plus PI analysis after treatment for 24 h. Data presented as mean ± SEM, *n* = 3, *P*‐values are calculated using one‐way ANOVA with Dunnett correction, *P < 0.05, **P < 0.01, ***P < 0.001.

Increased copy number and mRNA expression levels of *TACR1* (encoding for NK‐1R protein) were observed in the two colon cancer datasets (**Figure** **1**A), suggesting the potential role of the SP/NK‐1R signaling pathway in the pathogenesis of CRC. To further evaluate the prognostic potential of NK‐1R in CRC, we collected paraffin‐embedded tumor tissue blocks from 50 patients with colon cancer (Table S1, Supporting Information) and performed immunohistochemical staining. Positive immunostaining for NK‐1R protein was detected in the cytoplasm of colon cancer cells but not in the stroma (Figure 1B). Approximately 50% (25/50) of patients showed a positive NK‐1R expression in the colon tumor tissues, while there was lack of signals in the matched non‐cancerous mucosa. Further analysis of the associations between NK‐1R expression and clinicopathological characteristics of 50 colon cancer patients revealed a significant correlation between NK‐1R protein expression and tumor site (*P* = 0.024), tumor grade (*P* = 0.023), and tumor‐nodes‐metastasis (TNM) stage (*P* = 0.004) (Table S2, Supporting Information). The positive NK‐1R immunostaining was more frequently observed in the tumors of the right‐sided colon, with poor grade, and in an advanced stage (Table S2, Supporting Information). Importantly, patients with NK‐1R‐positive colon cancer presented a significantly poorer overall survival than those with NK‐1R‐negative colon cancer (*P* = 0.004) (Figure 1C). The NK‐1R protein expression level was also measured in five human CRC cell lines (SW620, HCT116, SW480, HT29, and RKO) and in normal colorectal tissues collected from six patients (Figure 1D,E). The NK‐1R protein expression was undetectable in normal colorectal tissues but increased significantly in human CRC cell lines, strongly supporting the oncogenic role of NK‐1R in CRC.

To characterize the functional role of NK‐1R in CRC, we assessed the viability of CRC cells after treatment with the NK‐1R antagonist SR140333 and aprepitant.^[^ ^11^ ^]^ Both antagonists potently inhibited cell proliferation in five human CRC cell lines in a dose‐ and time‐dependent manner (Figure 1F and Appendix A1 and Figure S1, Supporting Information). Alternatively, depletion of NK‐1R by short hairpin RNA (shRNA) in HCT116 and SW620 cells significantly reduced the cell proliferation (Figure 1G).

The reduced cell viability in the presence of NK‐1R antagonists was achieved by cell cycle arrest at the G_0_/G_1_ phase (Figure 1H and Appendix A1 and Figure S2A, Supporting Information) and induction of apoptotic cell death (Figure 1I and Appendix A1 and Figure S2C, Supporting Information). Disruption of cell cycle progression is associated with decreased cyclin D1, cyclin‐dependent kinase (CDK) 4, cyclin B1, and CDK1 protein levels and an increase in the expression of CDK inhibitors p15 and p21 (Appendix A1 and Figure S2B, Supporting Information). In line with a previous study on blocking NK‐1R in inducing apoptosis in human SW403 colon carcinoma cell line,^[^ ^10c^ ^]^ HCT116 and SW620 cells displayed typical apoptotic characteristics such as chromosome condensation, nuclear fragmentation, and membrane blebbing in the presence of SR140333 (Appendix A1 and Figure S3, Supporting Information). Annexin V and propidium iodide (PI) staining assays showed that the proportion of cells distributed in the areas of early apoptosis (Annexin V positive, Q4 area) and late apoptosis (Annexin V and PI double positive, Q2 area) was significantly increased in a dose‐dependent manner after exposure to SR140333 (Figure 1I and Appendix A1 and Figure S2C, Supporting Information). Moreover, treatment with SR140333 resulted in an increase in the abundance of proapoptotic proteins including BCL2‐associated X (BAX), cleaved Caspase 3, and cleaved poly (ADP‐Ribose) polymerase 1 (PARP1), and a decrease in the expression of antiapoptotic protein B‐cell CLL/Lymphoma 2 (BCL‐2)(Appendix A1 and Figure S2D, Supporting Information), indicating that blocking NK‐1R activates the intrinsic apoptotic pathways in human CRC cells.

### 2.2 Inactivation of ERK1/2‐c‐Myc Signaling Mediating NK‐1R Antagonist‐Induced Apoptosis in Human CRC Cells

**Figure 2:**
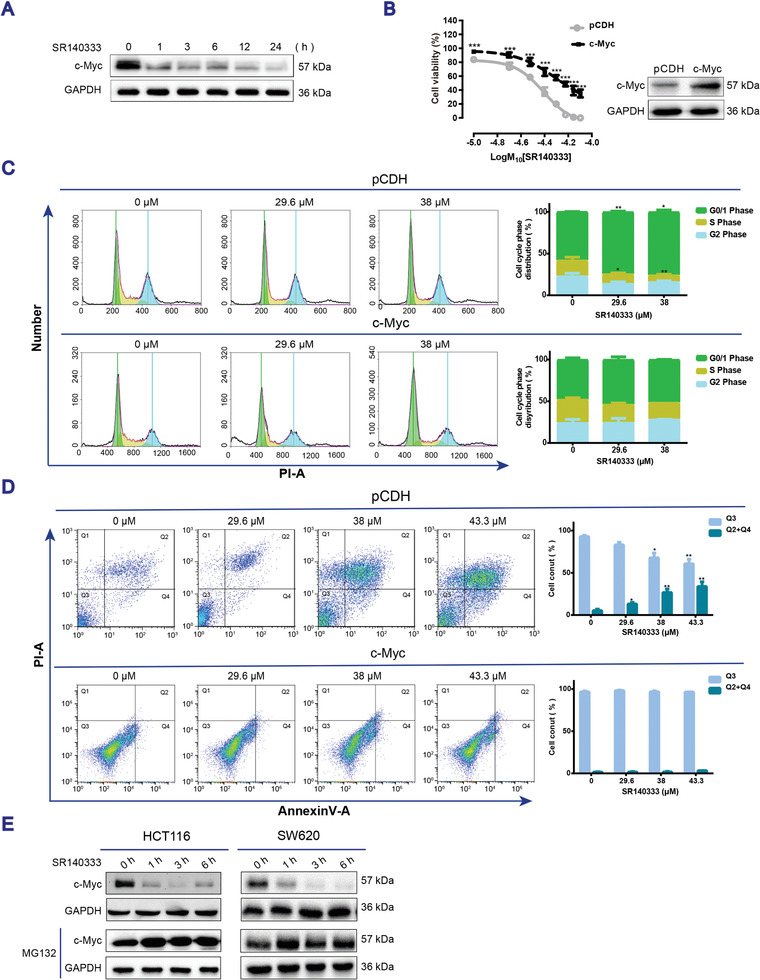
Decrease of c‐Myc mediates cytotoxicity of SR140333 in human colon cancer cells. A) Western blotting of c‐Myc expression in HCT116 cells treated with 38 × 10^−6^ m SR140333 at the indicated time points. B) HCT116 cells were stably transfected with either empty vector pCDH or the vector expressing human c‐Myc and then treated with SR140333 at the indicated doses for 24 h. Cell viability was measured by trypan blue exclusion assay. Data are expressed as mean ± SEM, *n* = 3. ***P < 0.001 by student's *t‐*test as compared with the cells transfected with empty vector. Western blotting of c‐Myc expression. GAPDH was used as a loading control. C,D) Cell cycle analysis and cell apoptotic analysis of HCT116 with or without c‐Myc overexpression treated with SR140333 for 24 h. Data presented as mean ± SEM, *n* = 3, *P*‐values are calculated using one‐way ANOVA with Dunnett correction, *P < 0.05, **P < 0.01, ***P < 0.001, compared with the vehicle control group. E) Western blotting of c‐Myc expression in cells pretreated with 10 × 10^−6^ m MG132 for one hour followed by treatment with SR140333 (38 × 10^−6^ m for HCT116 cells and 20.3 × 10^−6^ m for SW620 cells) at the indicated time points. Representative Western blotting images from at least three independent experiments were shown in (A)–(E).

We recently reported that blocking NK‐1R rapidly decreased the expression of c‐Myc in human myeloid leukemia cells.^[^ ^12^ ^]^ Garnier et al. also detected the downregulation of c‐Myc expression after treatment with aprepitant in human CRC cells using a reverse phase protein array.^[^ ^10a^ ^]^ Since c‐Myc is a master molecule of cell proliferation and survival,^[^ ^13^ ^]^ we investigated whether c‐Myc downregulation mediated the cytotoxicity caused by treatment with NK‐1R antagonists. Blocking NK‐1R with SR140333 resulted in a rapid decrease in c‐Myc protein expression in both HCT116 (**Figure** **2**A) and SW620 cells (Appendix A1 and Figure S4A, Supporting Information). The constitutive overexpression of c‐Myc in these two cell lines significantly increased the cell viability (Figure 2B and Appendix A1 and Figure S4B, Supporting Information), restored the cell cycle progression (Figure 2C and Appendix A1 and Figure S4C, Supporting Information), and protected the cells against apoptotic cell death (Figure 2D and Appendix A1 and Figure S4D, Supporting Information) in the presence of SR140333, confirming that c‐Myc mediates for the response to SR140333 in human CRC cells. Furthermore, reduction of c‐Myc protein expression upon SR140333 treatment was effectively inhibited by pretreatment with the proteasome inhibitor MG132 (Figure 2E), suggesting that the decrease in c‐Myc protein expression is attributed to the loss of c‐Myc protein stability.

**Figure 3:**
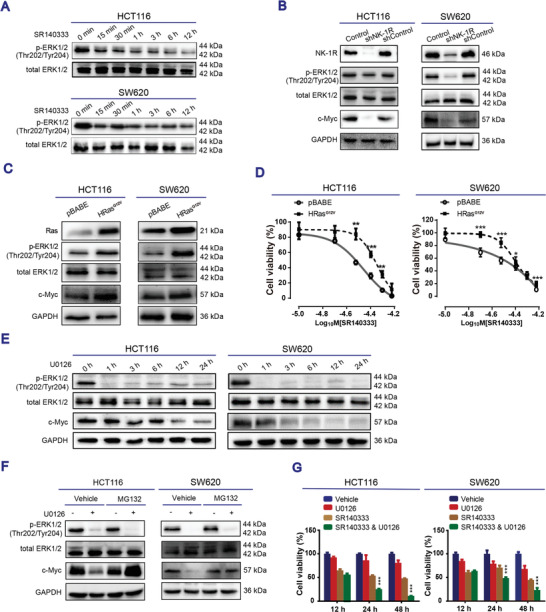
Blocking NK‐1R suppressed pERK1/2‐c‐Myc signaling pathway. A) Western blotting in HCT116 and SW620 cells treated with SR140333 at 38 and 20.3 × 10^−6^ m, respectively, as the indicated time points. B) Western blotting in HCT116 and SW620 cells after knockdown of NK‐1R by shRNA. C) Western blotting in cells stably transfected with either empty vector or the vector expressing HRAS^G12V^. D) HCT116 cells were stably transfected with either empty vector pBabe or the vector expressing HRAS^G12V^. Cell viability was measured by trypan blue exclusion assay. Data are expressed as mean ± SEM, *n* = 3. *P < 0.05, **P < 0.01, and ***P < 0.001 by student's *t‐*test as compared with the control group transfected with empty vector pBabe. E) Western blotting in cells treated with 10 × 10^−6^ m U0126 at the indicated time points. F) Western blotting in cells pretreated with 10 × 10^−6^ m MG132 for 1 h and then treated with 10 × 10^−6^ m U0126 for 3 h. G) Cell viability of cells pretreated with 10 × 10^−6^ m U0126 for 1 h and then treated with SR140333 (38 × 10^−6^ m for HCT116 cells and 20.3 × 10^−6^ m for SW620 cells), measured by MTT assay. Data presented as mean ± SEM, *n* = 6, *P*‐values are calculated using one‐way ANOVA with Tukey correction, **P < 0.01, ***P < 0.001, compared with the SR140333 treatment group. A–C,E,F) Representative Western blotting images from at least three independent experiments.

ERK signaling is a major pathway downstream of NK‐1R activation^[^ ^14^ ^]^ and regulates c‐Myc protein stability.^[^ ^15^ ^]^ Indeed, treatment with either SR140333 (**Figure** **3**A) or aprepitant (Appendix A1 and Figure S5, Supporting Information) resulted in an acute reduction in ERK1/2 phosphorylation, along with diminished c‐Myc protein expression in HCT116 and SW620 cells. Similar changes were observed when NK‐1R was depleted by shRNA (Figure 3B). Thus, we hypothesized that NK‐1R blockage reduced the c‐Myc protein stability through suppression of the ERK signaling pathway. Indeed, hyperactivation of the ERK signaling by expressing constitutively active harvey rat sarcoma viral oncogene homolog (HRAS)^G12V^ increased the c‐Myc protein abundance (Figure 3C) and critically protected the human CRC cells from SR140333‐induced cell death (Figure 3D), strongly supporting that ERK signaling mediates the response to NK‐1R antagonists in human CRC cells. Conversely, directly targeting the ERK signaling pathway with U0126, a highly selective inhibitor of MEK1/2,^[^ ^16^ ^]^ decreased the c‐Myc protein expression at a slower rate than the ERK1/2 phosphorylation (Figure 3E), supporting that c‐Myc is the downstream target of ERK signaling. Moreover, the proteasome inhibitor MG132 blocked the decrease in c‐Myc protein expression induced by treatment with U0126 (Figure 3F). Therefore, blocking the NK‐1R induces c‐Myc degradation by suppressing the RAS/ERK signaling. Treatment with SR140333 in combination with U0126 further reduced the cell viability compared with treatment with U0126 or SR140333 alone (Figure 3G), indicating that suppression of the ERK signaling increased the human CRC cells’ sensitivity to NK‐1R antagonist.

### 2.3 Blocking NK‐1R Inducing ER Calcium Release and ER Stress

**Figure 4:**
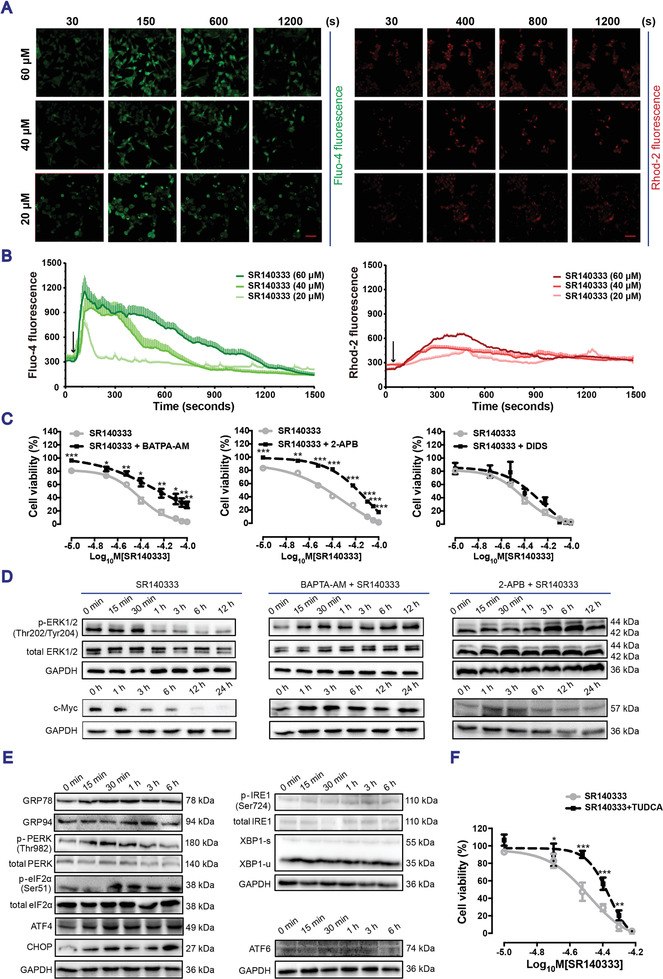
Blocking NK‐1R induces ER calcium release and ER stress in HCT116 cells. A) Images of intracellular calcium indicator Fluo‐4‐AM staining and mitochondrial calcium indicator Rhod‐2‐AM staining in HCT116 cells before and after treatment with SR140333 at 20 × 10^−6^, 40 × 10^−6^, and 60 × 10^−6^ m. B) The mean fluorescence intensity of 20 cells randomly picked up from at least two fields were calculated and presented as means ± SEM from at least three independent experiments. Arrow indicated the time of adding SR140333 after initial measurement for 50 s. C) HCT116 cells were pretreated with either BAPTA‐AM (10 × 10^−6^ m), 2‐APB (10 × 10^−6^ m), or DIDS (20 × 10^−6^ m) for 1 h, respectively, followed by SR140333 treatment for 24 h. Cell viability was measured by trypan blue exclusion assay. Data presented as mean ± SEM, *n* = 3, *P*‐values are calculated using student's *t*‐test, *P < 0.05, **P < 0.01, ***P < 0.001. D) Western blotting in HCT116 cells treated with SR140333 (38 × 10^−6^ m) for 24 h after pretreatment with BAPTA‐AM (10 × 10^−6^ m) or 2‐APB (10 × 10^−6^ m) for 1 h. E) Western blotting of ER stress markers in HCT116 cells treated with SR140333 at 38 × 10^−6^ m. GAPDH was used as a loading control. F) The viability of HCT116 cells treated with SR140333 alone or in combination with the ER stress inhibitor TUDCA (20 × 10^−6^ m) for 24 h as measured by MTT. Data presented as mean ± SEM, *n* = 3, *P*‐values are calculated using student's *t*‐test, *P < 0.05, **P < 0.01, ***P < 0.001. A,B,D,E) Representative Western blotting images from at least three independent experiments.

Activation of the NK‐1R signaling has been linked to intracellular calcium mobilization. We recently reported that NK‐1R antagonists promote rapid cytosolic and mitochondrial calcium mobilization in human myeloid leukemia.^[^ ^12^ ^]^ Therefore, we examined the extent of calcium mobilization upon modulation of NK‐1R activity in human CRC cells. Fluo‐4 and Rhod‐2 fluorescent probes were used to measure the cytosolic and mitochondrial free calcium levels, respectively. As observed in human myeloid leukemia,^[^ ^12^ ^]^ stimulation of NK‐1R with SP did not markedly affect either the cytosolic calcium flux or the mitochondrial calcium flux (Appendix A1 and Figure S6A–D, Supporting Information). By contrast, blocking the NK‐1R with SR140333 induced a rapid and potent cytosolic calcium elevation followed by a transient and weak increase in mitochondrial calcium flux in a dose‐dependent manner in both HCT116 cells (**Figure** **4**A,B) and SW620 cells (Appendix A1 and Figure S7A,B, Supporting Information). To evaluate the functional role of calcium signaling in NK‐1R blockade‐induced cell death, the cells were pretreated with either the calcium chelating reagent 1,2‐bis(o‐aminophenoxy)ethane‐N,N,N′,N′‐tetraacetic acid (BAPTA) or the inositol 1,4,5‐triphosphate receptor (IP_3_R) inhibitor 2‐Aminoethoxydiphenyl borate (2‐APB), which blocks calcium release from the ER.^[^ ^12^, ^17^ ^]^ Both BAPTA and 2‐APB increased the cell viability in the presence of SR140333, supporting the hypothesis that cytosolic calcium flux contributes to the SR140333‐induced cell apoptosis (Figure 4C and Appendix A1 and Figure S7C, Supporting Information). In contrast to myeloid leukemia cells, which showed an ER‐mitochondrial calcium flux,^[^ ^12^ ^]^ human colon cancer cells did not show a robust and consistent mitochondrial calcium peak after treatment with SR140333. Moreover, treatment with 4,4′‐diisothiocyanatostilbene‐2,2′‐disulfonic acid disodium salt hydrate (DIDS), an inhibitor of voltage‐dependent anion channel type 1 in the outer mitochondrial membrane involved in the mitochondrial calcium flux, did not rescue the cell death induced by SR140333 (Figure 4C and Appendix A1 and Figure S7C, Supporting Information). Therefore, ER‐mitochondrial calcium fluxes do not likely contribute to the occurrence of SR140333 cytotoxicity. Strikingly, both BAPTA and 2‐APB prevented the decrease in ERK1/2 activity and c‐Myc expression after SR140333 treatment (Figure 4D and Appendix A1 and Figure S7D, Supporting Information), suggesting the regulation of ERK‐c‐Myc signaling via ER calcium release upon NK‐1R blockade.

Calcium release is a key feature of ER stress, and disruption of ER function and accumulation of unfolded proteins in the ER lumen result in the activation of an unfolded protein response (UPR).^[^ ^18^ ^]^ Therefore, we hypothesized that blocking NK‐1R in human colon cancer cells will leads to the development of ER stress. In mammals, there are three classes of ER stress sensors: inositol‐requiring enzyme 1*α* (IRE1*α*) and IRE1*β*, PERK, and activating transcription factor 6 (ATF6; both *α* and *β* isoforms).^[^ ^19^ ^]^ Increased expression levels of GRP78, GRP94, p‐PERK, p‐eIF2*α*, ATF4, and CHOP, but not those of p‐IRE1, XBP1‐s, and ATF6, upon treatment with SR140333 (Figure 4E and Appendix A1 and Figure S7E, Supporting Information) or aprepitant (Appendix A1 and Figure S8, Supporting Information) in both HCT116 and SW620 cells showed that NK‐1R blockage activates ER stress through the PERK‐ATF4 arm of the UPR. Importantly, the ER stress inhibitor tauroursodeoxycholate (TUDCA)^[^ ^20^ ^]^ rescued cell viability after SR140333 treatment (Figure 4F and Appendix A1 and Figure S7F, Supporting Information), confirming the ER stress‐mediated sensitivity to NK‐1R antagonists.

We recently reported that blocking NK‐1R induces oxidative stress in patients with human myeloid leukemia.^[^ ^12^ ^]^ However, both HCT116 and SW620 cells treated with SR140333 did not exhibit mitochondrial reactive oxygen species (ROS) or cytosolic ROS accumulation (Appendix A1 and Figure S9, Supporting Information), indicating the cell‐type‐dependent molecular effects of NK‐1R antagonists. Taken together, our data reveal that NK‐1R antagonists induce apoptosis in human colon cancer cells by suppressing the ERK‐c‐Myc signaling via ER calcium release and activation of PERK‐ATF4‐CHOP ER stress signaling pathways.

### 2.4 Blocking NK‐1R Inhibiting Human Colon Cancer Xenograft Growth

**Figure 5:**
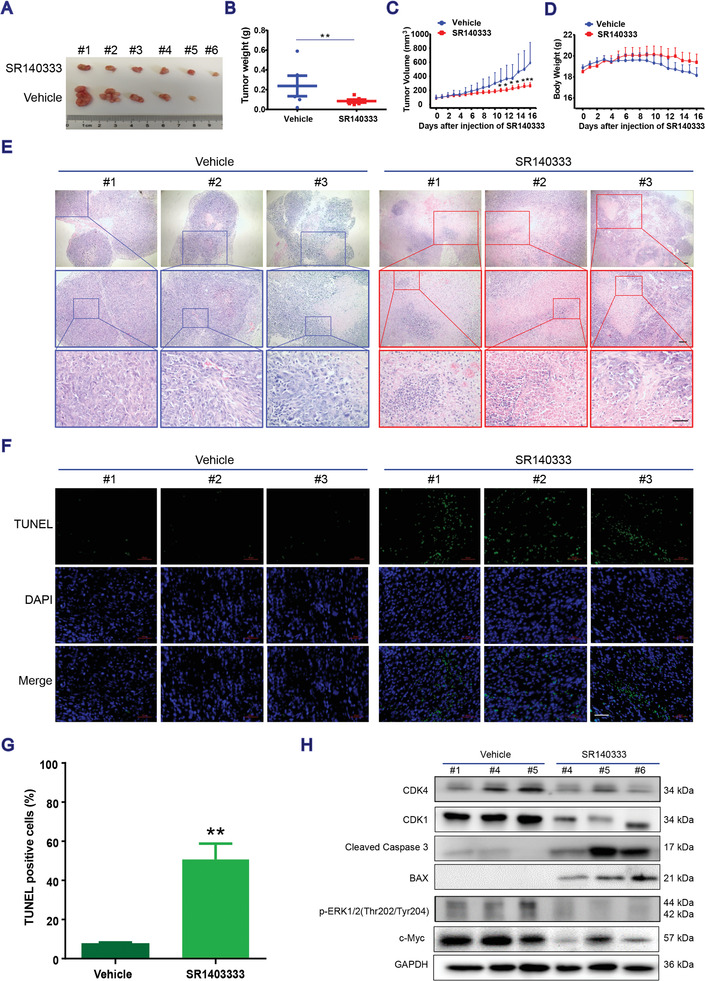
Blocking NK‐1R inhibits human CRC xenograft growth. A) The images of tumors excised from all nude mice on day 16 (*n* = 6 mice for SR140333 group and *n* = 5 mice for vehicle group). B) Tumor mass, C) tumor volume, and D) body weight were measured in HCT116 xenografts. Data presented as mean ± SEM, *P*‐values are calculated using student's *t*‐test, *P < 0.05, **P < 0.01, ***P < 0.001, compared with the vehicle group. E) H&E‐staining of tumor tissues. Scale bar, 50 µm. F) TUNEL staining. Scale bar, 50 µm. G) The percentage of TUNEL positive cells was determined by the cell numbers of TUNEL positive/the total numbers. Data presented as mean ± SEM, *n* = 3, *P*‐values are calculated using student's *t*‐test, **P < 0.01. H) Western blotting in HCT116 xenografts. Representative images from at least three independent experiments.

We then evaluated the efficacy of the NK‐1R antagonist SR140333 in human colon cancer xenografts. SR140333 was administered at 10 mg kg^−1^ once daily via peritumoral injection for 16 d in BALB/c nude mice with HCT116 xenografts. SR140333 treatment significantly decreased the tumor size, tumor weight, and tumor growth rate compared with the vehicle (**Figure** **5**A–C) without loss of body weight (Figure 5D). Hematoxylin and eosin (H&E)‐stained xenograft tissue sections showed reduction in the intensity of nuclei staining in the SR140333 group (Figure 5E), supporting the inhibition of HCT116 xenograft growth by SR140333 treatment. SR140333‐induced apoptosis in vivo was confirmed by positive terminal deoxynucleotidyl transferase dUTP nick end labeling (TUNEL) staining of tumor tissues (Figure 5F,G), accompanied by an increase in proapoptotic proteins cleaved Caspase 3 and BAX and a decrease in cell cycle‐associated proteins CDK4 and CDK1 in SR140333‐treated mice bearing HCT116 xenografts (Figure 5H). The critical role of ERK‐c‐Myc signaling in the antitumor effect of the NK‐1R antagonist was further confirmed in vivo, as evidenced by the suppression of ERK1/2 phosphorylation and c‐Myc protein expression in the tumors of the SR140333 treatment group (Figure 5H).

### 2.5 Blocking NK‐1R Increasing the Sensitivity of CRC Cells to Chemotherapeutic Drugs and Overcoming Resistance to Chemotherapy In Vitro and In Vivo by Inducing ER Stress and Suppressing the ERK‐c‐Myc Signaling

Chemotherapy is one of the conventional treatments for CRC, along with resection and radiotherapy. Although 5‐fluorouracil (5‐FU), irinotecan (SN‐38 as its active metabolite), and oxaliplatin as first‐line chemotherapeutic agents effectively improve the survival of CRC patients, the serious side effects and development of resistance are obstacles to the clinical effectiveness of chemotherapy.^[^ ^21^ ^]^ By analyzing the CRC cell line datasets from the Oncomine, the mRNA expression levels and copy number of *TACR1* (which encodes NK‐1R) and *TAC1* (which encodes SP) were significantly elevated in the CRC cells resistant to a series of chemotherapeutic agents compared with the sensitive cells (Appendix A1 and Figure S10A–E, Supporting Information), suggesting that the SP/NK‐1R signaling may contribute to the development of chemoresistance.

**Figure 6:**
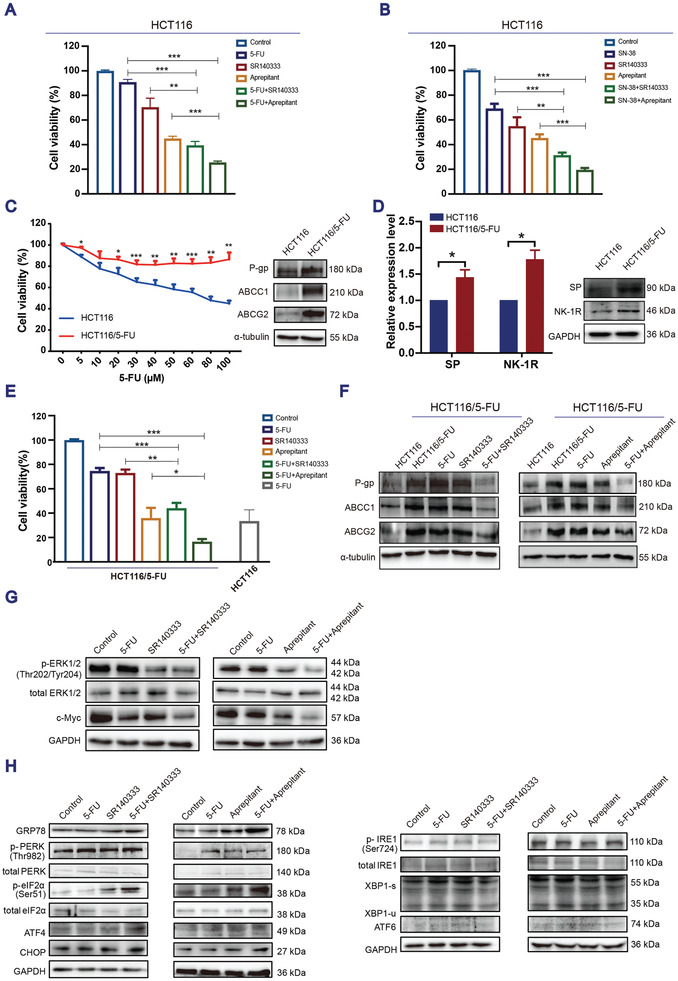
Blocking NK‐1R enhances the sensitivity of HCT116 cells to chemotherapeutic drugs and overcomes drug‐resistance in HCT116/5‐FU cells through ER stress‐mediated degradation of c‐Myc. A) Cell viability upon treatment with 5‐FU (60 × 10^−6^ m), SR140333 (30 × 10^−6^ m), or Aprepitant (40 × 10^−6^ m) either as single agents or in combination at 24 h in HCT116 cells. Data presented as mean ± SEM, *n* = 3, *P*‐values are calculated using one‐way ANOVA with Tukey correction, ***P < 0.001. B) Cell viability upon treatment with SN‐38 (400 × 10^−9^ m), SR140333 (30 × 10^−6^ m), or Aprepitant (40 × 10^−6^ m) either as single agents or in combination at 24 h in HCT116 cells. Data presented as mean ± SEM, *n* = 3, *P*‐values are calculated using one‐way ANOVA with Tukey correction, *P < 0.05, **P < 0.01, ***P < 0.001. C) Cell viability of 5‐FU treatment for 24 h. Data presented as mean ± SEM, *n* = 3, *P*‐values are calculated using student's *t*‐test, *P < 0.05, **P < 0.01, ***P < 0.001. Representative Western blotting images from at least three independent experiments. D) Western blotting of SP and NK‐1R proteins in HCT116/5‐FU cells and HCT116 cells. Data presented as mean ± SEM, *n* = 3, *P*‐values are calculated using student's *t*‐test, *P < 0.05. E) Cell viability upon treatment with 5‐FU (80 × 10^−6^ m), SR140333 (30 × 10^−6^ m), or Aprepitant (40 × 10^−6^ m) at 48 h in HCT116/5‐FU cells compared with the viability of HCT116 cells treated with 5‐FU at 80 × 10^−6^ m. Data presented as mean ± SEM, *n* = 3, *P*‐values are calculated using one‐way ANOVA with Tukey correction, *P < 0.05, ***P < 0.001. F) Western blotting of drug‐resistant proteins in HCT116/5‐FU cells treated with 5‐FU (80 × 10^−6^ m), SR140333 (30 × 10^−6^ m), or Aprepitant (40 × 10^−6^ m) at 48 h. G) Western blotting of ERK‐c‐Myc signaling and H) ER stress pathways in HCT116/5‐FU cells treated with 5‐FU (60 × 10^−6^ m) with SR140333 (30 × 10^−6^ m) or Aprepitant (40 × 10^−6^ m) either as single agents or in combination at 24 h. GAPDH was used as a loading control. All the western blotting images are representative of at least three independent experiments.

Since NK‐1R antagonists exert a potent antitumor action through distinct mechanisms from chemotherapeutic drugs, we hypothesized that use of a combination of NK‐1R antagonists will improve its efficacy and reduce the incidence of chemotherapy‐induced toxicity in patients with colon cancer. Both 5‐FU and SN‐38 inhibited the proliferation of HCT116 and SW620 cells in a dose‐ and time‐dependent manner (Appendix A1 and Figure S11A, Supporting Information). The combination of NK‐1R antagonists SR140333 or aprepitant with 5‐FU or SN‐38 significantly decreased the cell viability compared with the use of a single agent (**Figure** **6**A,B and Appendix A1 and Figure S11B,C, Supporting Information).

To investigate whether NK‐1R antagonists can circumvent the acquired chemotherapy resistance, we examined the efficacy of NK‐1R antagonists in HCT116 cells resistant to 5‐FU (HCT116/5‐FU cells). Compared with the parental HCT116 cells, the HCT116/5‐FU cells showed reduced drug response, as evidenced by increased cell viability in the presence of 5‐FU (Figure 6C) with elevated expression of classical proteins associated with chemotherapy resistance, including P‐glycoprotein (P‐gp), multidrug resistance‐associated protein 1 (ABCC1), and breast cancer resistance protein (ABCG2), compared with the sensitive cells (Figure 6C). An increase in SP and NK‐1R expression levels was detected in the HCT116/5‐FU cells (Figure 6D), suggesting that the SP/NK‐1R signaling may mediate resistance to 5‐FU in colon cancer cells. Strikingly, treatment with the NK‐1R antagonist SR140333 or aprepitant significantly increased the sensitivity of HCT116/5‐FU cells to 5‐FU, to a level comparable to that of the parental cells (Figure 6E). This improved response to 5‐FU was accompanied by decreased expression levels of P‐gp, ABCC1, and ABCG2 (Figure 6F). Importantly, suppression of p‐ERK and c‐Myc expression (Figure 6G) and upregulation of the PERK‐ATF4 ER stress signaling pathway were also detected in the HCT116/5‐FU cells treated with either SR140333 or aprepitant and further enhanced by combined treatment with 5‐FU (Figure 6H).

**Figure 7:**
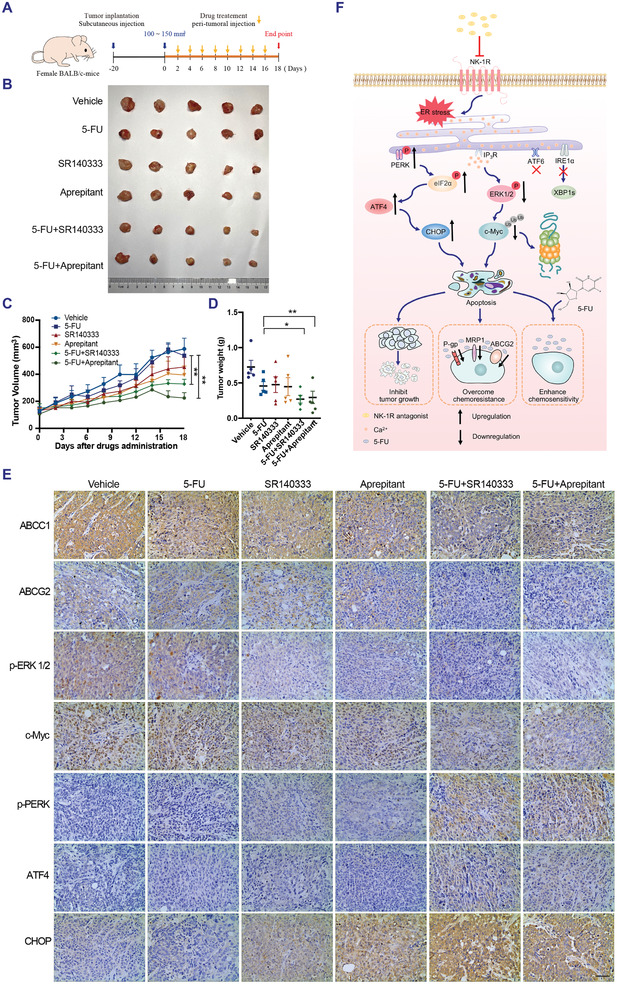
Blocking NK‐1R overcomes drug‐resistance in mice xenografted with HCT116/5‐FU cells in vivo through ER stress and suppression of ERK‐c‐Myc signaling. A) Schematic diagram showing treatment of HCT116 xenografts resistant to 5‐FU (HCT116/5‐FU) with 5‐FU (10 mg kg^−1^) combined with SR140333 (10 mg kg^−1^) or Aprepitant (10 mg kg^−1^). B) Photograph of isolated tumor xenografts at day 18 in each group. C) Tumor volumes. D) Tumor weights. Data presented as mean ± SEM, *n* = 5, *P*‐values are calculated using student's *t*‐test, *P < 0.05, **P < 0.01. E) IHC staining. Scale bar: 50 µm. F) Model of NK‐1R antagonist inducing apoptosis in CRC cells through activation of ER stress pathway and suppression of ERK‐c‐Myc signaling via ER calcium release and consequently inhibiting tumor growth, enhancing chemosensitivity, as well as overcoming chemoresistance.

To evaluate the therapeutic potential of a combination NK‐1R antagonists to overcome chemotherapy resistance in vivo, we subcutaneously inoculated HCT116/5‐FU cells into nude mice and then treated them with NK‐1R antagonists SR140333 or aprepitant either as a single agent or in combination with 5‐FU, as shown in **Figure** **7**A. A marked reduction in the tumor size, growth rate, and tumor weight of HCT116/5‐FU cell xenografts was observed in the combination therapy groups compared with the 5‐FU group (Figure 7B–D and Appendix A1 and Figure S12, Supporting Information). No significant changes were observed in the general appearance, body weight, organ index, or microanatomy of organs among the five groups (Appendix A1 and Figures S13 and S14A–C, Supporting Information).

We further characterized the molecular mechanisms underlying the improved efficacy of the combination treatment in vivo. In agreement with the in vitro results, the combination of SR140333 or aprepitant and 5‐FU decreased the expression levels of ABCC1, ABCG2, p‐ERK1/2, and c‐Myc and increased those of p‐PERK, ATF4, and CHOP compared with 5‐FU treatment alone (Figure 7E and Appendix A1 and Figure S15, Supporting Information). Collectively, these results showed that treatment with NK‐1R antagonists overcame the resistance to 5‐FU through suppression of the ERK‐c‐Myc signaling and activation of the PERK‐ATF4 ER stress signaling pathways.

## 3 Discussion

It is now becoming clear that SP/NK‐1R signaling is involved in cancer pathogenesis and promotes proliferation, angiogenesis, and metastasis of cancer cells, which has been well summarized in several reviews.^[^ ^4^, ^22^ ^]^ Therefore, NK‐1R antagonists can be used as a novel approach for cancer treatment. Aprepitant, an NK‐1R antagonist, has been approved by the Food and Drug Administration for the treatment of nausea and vomiting caused by cancer chemotherapy^[^ ^23^ ^]^ and thus can be directly used to test the antitumor action in clinical trials. Small molecules, including peptide antagonists and nonpeptide antagonists, have demonstrated a broad spectrum of antitumor activity in various types of cancer. The growth inhibitory effect is not dependent on the chemical structures,^[^ ^22b^ ^]^ but specifically occurs through NK‐1R in cancer cells highly expressing this receptor in a dose‐dependent manner.^[^ ^6^, ^10^ ^]^ Notably, a case report revealed that treatment with a combination of radiotherapy and aprepitant (compassionate use 1140 mg d^−1^) in a patient with lung cancer for 45 d led to the disappearance of the tumor mass without the occurrence of side effects.^[^ ^24^ ^]^ Moreover, the safety of aprepitant has been demonstrated in various experimental models.^[^ ^10^, ^22^ ^]^ The dose of aprepitant used in the treatment of patients with cancer (35–50 mg kg^−1^ d^−1^) would be much lower than the carcinogenic doses detected in rodents (125–2000 mg kg^−1^ d^−1^).^[^ ^25^ ^]^ This safety and tolerability are crucial for repurposing aprepitant as an anticancer drug because it has been suggested to be administered at high doses (>20 mg kg^−1^ d^−1^) daily for an extended period of time to achieve an antitumor effect in contrast to the current antiemetic treatment (120, 80, and 80 mg for three consecutive days).^[^ ^10^, ^22^ ^]^ Moreover, the specificity of the antitumor action and safety of aprepitant enable the development of combination therapies for the treatment of cancer to reduce the undesirable side effects of conventional radiotherapy and chemotherapy.

In this study, we confirmed that the inhibition of the NK‐1R‐induced apoptosis in human colon cancer cells was in accordance with the findings of other research groups.^[^ ^10a,c^ ^]^ This study was the first to demonstrate the in vivo efficacy of NK‐1R antagonist in inhibiting tumor growth and improving the efficacy of chemotherapy by overcoming chemoresistance in CRC xenograft mouse models. We performed an in‐depth mechanistic analysis to characterize the molecular changes that occur upon NK‐1R blocking in CRC cells. We found that ER stress mediates the cytotoxicity of NK‐1R antagonists in CRC cells (Figure 7F). The ER is involved in multiple cellular processes, such as protein processing, lipid synthesis, and calcium storage and release. Disruption of ER homeostasis induces an ER stress response or an unfolded protein response. Cancer cells exhibit activation of the ER stress response, which is considered a prosurvival adaptive response that promotes tumor formation, progression, and chemoresistance.^[^ ^26^ ^]^ ER stress addiction represents a vulnerability with potential applications in cancer treatment by either inhibiting ER stress response to abrogate the prosurvival mechanism or exacerbate ER stress to activate proapoptotic signaling.^[^ ^27^ ^]^ We demonstrated that blocking the NK‐1R induced a sustained ER stress, which leads to apoptotic cell death through ER calcium release followed by suppression of the ERK‐c‐Myc signaling and activation of the PERK‐eIF2a‐ATF4‐CHOP ER stress pathway (Figure 7F).

Suppression of the ERK‐c‐Myc signaling via ER calcium release is a novel mechanism that mediates the response to NK‐1R antagonists in CRC. c‐Myc is a master regulator of cell proliferation and survival^[^ ^13^ ^]^ and is integrated into ER stress in cancer cells.^[^ ^26b^ ^]^ In this study, we demonstrated that c‐Myc is a key player in NK‐1R antagonist‐induced cell death. Although a decrease in the c‐Myc expression upon NK‐1R inhibition was proposed as a downstream effect of the suppression of the Wnt signaling,^[^ ^10a^ ^]^ we discovered that the degradation of c‐Myc protein upon NK‐1R antagonist treatment was made possible through the inhibition of ERK signaling, an important regulatory mechanism of c‐Myc expression reported previously.^[^ ^15^, ^28^ ^]^

Activation of the ERK signaling by the NK‐1R has been linked to the assembly of an endosomal signaling complex (signalosome), which mediates sustained intracellular signaling.^[^ ^29^ ^]^ Upon SP binding to the NK‐1R, several signaling proteins including *β*‐arrestin, Src, MAPK/ERK kinase kinase (MEKK), and ERK1/2 were recruited to internalize NK‐1R and form endosomes that promote ERK1/2 phosphorylation and translocation to the nucleus.^[^ ^29^, ^30^ ^]^ In this study, we demonstrated that treatment with NK‐1R antagonists induced acute calcium release from the ER and pharmaceutically blocked the ER calcium release, preventing a decrease in ERK signaling activity and c‐Myc protein expression (Figure 4D). This finding supports the strong connection between ER calcium release and ERK‐c‐Myc signaling. Although the activation of ERK signaling in response to ER stress is thought to promote adaptation, survival, and resistance to ER stress,^[^ ^31^ ^]^ we propose that inhibition of ERK signaling upon ER calcium release induced by treatment with NK‐1R antagonist abrogates its protective functions that attempt to restore ER homeostasis and consequently promotes cell death. Other mitogen‐activated protein kinase (MAPK) signaling pathways, including p38 and c‐Jun N‐terminal kinase, have been shown to cause ER stress‐induced cell death.^[^ ^27^ ^]^ Thus, the functional role of these MAPK signaling pathways in NK‐1R antagonist‐induced cell death must be investigated in the future. In addition to the suppression of ERK signaling, other mechanisms have been reported to mediate the proapoptotic effect of ER calcium release, such as activation of calcium/calmodulin‐dependent protein kinase II^[^ ^32^ ^]^ or disruption of mitochondrial structure/function by interfering with antiapoptotic molecules BCL‐2, BCL‐xL, and MCL1, resulting in cytochrome c release after fluxing into the mitochondria.^[^ ^27b^ ^]^ In addition to the ER calcium release, we also identified the specific activation of the PERK‐eIF2a‐ATF4‐CHOPER stress pathway in CRC cells treated with NK‐1R antagonists (Figure 4E). An increase in CHOP expression can activate the intrinsic apoptotic pathway by decreasing the expression of antiapoptotic molecules such as BCL‐2 and BCL‐xL and increasing the expression of proapoptotic molecules such as BAX and Bcl‐2 homologous antagonist/killer (BAK), as well as promoting calcium release.^[^ ^27c^ ^]^

A combination of NK‐1R antagonists with chemotherapy/radiotherapy was reported to reduce the side effects of conventional cancer therapy and improved the antitumor effects.^[^ ^24^, ^33^ ^]^ In this study, we demonstrated that treatment with NK‐1R antagonists not only induced apoptosis and inhibited tumor growth but also improved the efficacy of the chemotherapeutic drug 5‐fluorouracil in CRC. Interestingly, ER stress is involved in 5‐FU resistance in human colon cancer cells, and knockdown of ER stress‐related proteins GPR78 and ATF6 improved the chemosensitivity.^[^ ^34^ ^]^ In contrast to their findings, our in vitro (Figure 6H) and in vivo (Figure 7E) evidence showed that the NK‐1R antagonists overcame the 5‐FU resistance by further enhancing the PERK‐eIF2a‐ATF4‐CHOP ER stress signaling. We propose that treatment with a combination of NK‐1R antagonists may generate a sustained and severe ER stress condition and consequently initiate a proapoptotic signaling program, resulting in increased cell death. Not only in colon cancer presented in this study, we recently reported that blockage of NK‐1R in myeloid leukemia cells elicited a rapid ER calcium release, which mediates the cytotoxicity of NK‐1R antagonist.^[^ ^12^ ^]^ Therefore, it is likely that induction of ER stress is a key mechanism of action mediating the cytotoxicity of NK‐1R antagonists in tumor cells. This finding expands our understanding of targeting the NK‐1R‐mediated GPCR signaling in human colon cancer cells and may be applied as a predictive biomarker for evaluation of the efficacy of NK‐1R antagonists in clinical settings.

We demonstrated the in vitro and in vivo efficacy of NK‐1R antagonists in promoting cell apoptosis, inhibiting tumor growth, increasing sensitivity, and overcoming resistance to chemotherapy in CRC. More importantly, we found that ER stress mediates the cytotoxicity of NK‐1R antagonists by inducing ER calcium release, suppressing the ERK‐c‐Myc signaling, and activating the PERK‐eIF2a‐ATF4‐CHOP ER stress pathway. These novel mechanistic findings revealed the downstream molecular events targeting the NK‐1R‐mediated GPCR signaling in human CRC and will facilitate the rational design of clinical trials to test the application of NK‐1R antagonists for the treatment of CRC, especially for the treatment of CRC with chemotherapy resistance.

## 4 Experimental Section

### Cell Lines and Reagents

Fivehuman colon cancer cell lines, HCT116, HT29, SW480, SW620, and RKO, were obtained from the First Affiliated Hospital, Zhejiang University; HCT116 cells resistant to 5‐FU (HCT116/5‐FU) were obtained from BeNa Culture Collection Biotech Co., Ltd. SR140333 and aprepitant were synthetized by WuXi AppTec (China) and Sichuan Jinqianye Technology Co., Ltd. (China), respectively, and dissolved in dimethyl sulfoxide (DMSO, Sigma).^[^ ^35^ ^]^ SP was synthesized by Sangon Biotech Co., Ltd. with >98% purity. MG132 from Solarbio Life Science; U0126 and BAPTA from Topscience Co., Ltd.; 2‐APB, DIDS, TUDCA, 5‐FU, and SN‐38 from MedChemExpress were dissolved in DMSO to 10 × 10^−3^ m stock solution and stored at −20 °C. Details of the procedure are available in Appendix A1 in the Supporting Information.

### Plasmid Construction, Cell Proliferation Assay, Flow Cytometric Analysis, and Western Blotting

Details of plasmid construction and transfection are provided in Appendix A1 in the Supporting Information. The cells were seeded in 96‐well plates or in 12‐well plates and then harvested at the indicated time points after treatment to detect cell viability, cell cycle distribution, apoptosis, and western blotting as described in the previous publication.^[^ ^36^ ^]^ Details of the procedure are available in Appendix A1 in the Supporting Information.

### Calcium Mobilization Analysis and ROS Detection

The cells were seeded in a cover glass‐bottom dish (SPL Life Sciences Co., Ltd., Korea). Calcium mobilization analysis was performed using Fluo‐4 AM (acetoxymethyl) ester (Invitrogen, San Diego, CA, USA) or Rhod‐2 AM (Invitrogen, San Diego, CA, USA), as described in the recent report.^[^ ^12^ ^]^ Intracellular ROS were measured by flow cytometry using a cell‐based ROS assay kit (S0033; Beyotime Biotechnology, Beijing, China) and MitoSOX (M36008, Invitrogen, USA). Details of the procedure are available in Appendix A1 in the Supporting Information.

### Mouse Models and Patient Issues

The animal protocols were performed in accordance with the Guide for the Care and Use of Laboratory Animals and approved by the Animal Care and Use Committee of the Ethics Committee of Zhejiang Sci‐Tech University and Hangzhou Normal University. The HCT116 cells (5 × 10^6^) were injected into the flanks of 4‐week‐old female BALB/c nude mice. When tumors reached 100–150 mm^3^, the mice were divided randomly into two groups (SR140333 group: *n* = 6 mice; vehicle group: *n* = 5 mice). A peritumoral injection was performed in each group once daily for 16 d. H&E staining, TUNEL staining, and western blotting were carried out to analyze the resected tumors.

The colon tissue samples were collected after obtaining the patient informed consent and the protocol was approved by the Institutional Review Board of the 903rd Hospital of The People's Liberation Army (PLA). The tissues of 50 colon cancer patients were collected from 2012 to 2015 at the 903rd Hospital of PLA, Hangzhou, China, for immunohistochemical analysis. The signal was quantified using the Allred score system, which represents the estimated proportion of positively stained cells combined with staining intensity, as previously described.^[^ ^37^ ^]^ Details of the procedure are available in Appendix A1 in the Supporting Information.

### Statistical Analysis

Each experiment was performed in triplicate. Statistical analyses were performed using GraphPad Prism version 8. Data were expressed as mean ± standard error of the mean (SEM). Student's *t*‐test was used to compare the differences between the two groups. For comparison between multiple groups with one control, statistical significance was determined by one‐way analysis of variance (ANOVA) followed by Dunnett's test. For other comparisons between multiple groups, statistical significance was determined using one‐way ANOVA followed by Tukey's test. *P* values of * < 0.05, ** < 0.01, and *** < 0.001 were considered significant.

## Conflict of Interest

The authors declare no conflict of interest.

## Author Contributions

Y.S., X.W., Y.M., and J.M. contributed equally to this work. C.F. conceived the study. C.F., J.K., and Y.C. designed the experiments. C.F., Y.S., X.W., Y.M., J.M., Q.Z., G.S., L.W., X.C., X.H., Y.W., and Z.Y. performed the experiments and analyzed the data. C.F., J.K., and Y.C. wrote the paper.

## Supporting information

Supporting Information

## Data Availability

Research data are not shared.
