# Supplementary material for: A Novel Mechanism of Endoplasmic Reticulum Stress‐ and c‐Myc‐Degradation‐Mediated Therapeutic Benefits of Antineurokinin‐1 Receptor Drugs in Colorectal Cancer
Source: Adv Sci (Weinh). 2021 Oct 3;8(21):2101936. doi: 10.1002/advs.202101936 (PMC8564433; doi:10.1002/advs.202101936)
Supplement: Supplementary file 1 — Supporting Information [file ADVS-8-2101936-s001.pdf]

© 2021 Wiley-VCH GmbH

ADVANCED SCIENCE
Open Access

Supporting Information

for *Adv. Sci.,* DOI: 10.1002/advs.202101936

A novel mechanism of endoplasmic reticulum stress- and c-Myc-degradation-mediated therapeutic benefits of anti-neurokinin-1 receptor drugs in colorectal cancer

*Yue Shi, Xi Wang, Yueming Meng,Junjie Ma, Qiyu Zhang, Gang Shao, Lingfei Wang, Xurui Cheng, Xiangyu Hong, Yong Wang, Zhibin Yan, Yihai Cao, Jian Kang, and Caiyun Fu**

A novel mechanism of endoplasmic reticulum stress- and c-Myc-degradation-mediated therapeutic benefits of anti-neurokinin-1 receptor drugs in colorectal cancer

Yue Shi,[1, #] Xi Wang,[2, #] Yueming Meng,[1,#] Junjie Ma,[1, #] Qiyu Zhang,[1] Gang Shao,[2] Lingfei Wang,[2] Xurui Cheng,[1] Xiangyu Hong,[1] Yong Wang,[1] Zhibin Yan,[1] Yihai Cao,[3] Jian Kang,[4,5] and Caiyun Fu[1,*]

Contents

Supplemental methods 3
Supplemental Figures 13
Figure S1 Blocking NK-1R by SR140333 inhibited cell proliferation in human colon cancer cells 13
Figure S2 Blocking NK-1R induced cell cycle arrest and apoptosis in human colon cancer cells. 14
Figure S3 Blocking NK-1R induced apoptosis in human colon cancer cells 15
Figure S4 Decrease of c-Myc mediates cytotoxicity of SR140333 in human colon cancer cells 16
Figure S5 Aprepitant suppresses p-ERK1/2-c-Myc signaling pathway 17
Figure S6 Stimulation of NK-1R with SP in HCT116 and SW620 cells did not affect cytosolic and mitochondrial calcium flux 18
Figure S7 Blocking NK-1R induces ER calcium release and ER stress in SW620 cells 20
Figure S8 Aprepitant induces ER stress 22
Figure S9 SR140333 did not affect reactive oxygen species production in HCT116 and SW620 cells 23
Figure S10 The mRNA expression and copy number levels of TACR1 and TAC1 in drug-sensitive and drug-resistant CRC cell lines in the Oncomine database. 24
Figure S11 Blocking NK-1R enhances the sensitivity of CRC cells to chemotherapeutic drugs 26
Figure S12 H&E staining of the tumor in mice treated with vehicle or Chemotherapy drugs. 27
Figure S13 The body weights of mice with HCT116/5-FU xenografts following treatment. 28
Figure S14 Chemotherapy drugs have no obvious toxic effects on mice 29
Figure S15 Quantification of positive immunohistochemistry staining in the tissue samples from HCT116/5-FU xenografts. 30
Table S1 The information of colon cancer patients. 31
Table S2 Associations of NK-1R expression with clinicopathological characteristics of 50 patients with colon cancer. 33

Supplemental methods

Data Mining in Oncomine. Gene copy number and expression data of patients and cell lines were extracted from Oncomine database (www.oncomine.org). *TACR1* copy number levels were included in TCGA Colorectal 2 and Barretina CellLine 2 (GSE36138) datasets by reporter 02-072503625. *TACR1* mRNA expression levels were included in Skrzypczak Colorectal (GSE20916), Garnett CellLine and Barretina CellLine (GSE36133) datasets by reporter 208048_at. *TAC1* copy number levels were included in Barretina CellLine 2 (GSE36138) dataset by reporter 07-097203463. *TAC1* mRNA expression levels were included in Garnett CellLine dataset by reporter 206552_s_at.

Cell culture. Five human CRC cell lines used in our investigation including HCT116, HT29, SW480, SW620 and RKO were obtained from the First Affiliated Hospital, Zhejiang University, and HCT116 cells resistant to 5-FU (HCT116/5-FU) was obtained from BeNa Culture Collection Biotech Co., Ltd. SW620 was cultured in DMEM (Life Technologies, Carlsbad, USA), RKO, HCT116, HT29, SW480, HCT116/5-FU were cultured in RIPM 1640 (Life Technologies, Carlsbad, USA). All the media were supplemented with 10% heat-inactivated FBS (fetal bovine serum, Solarbio Life Science, Beijing, China). All the cell lines were cultured at 37°C in a humidified atmosphere with 5% CO_2.

Drug treatment. SR140333 was synthetized by WuXi AppTec (China) and dissolved in DMSO (dimethyl sulphoxide, sigma). Aprepitant was synthetized by Sichuan Jinqianye Technology Co., Ltd. (China) and dissolved in DMSO. SP was synthetized by Sangon Biotech Co., Ltd. with >98% purity and dissolved in sterile water. Proteasome inhibitor MG132 from Solarbio Life Science (Beijing, China), MEK1/2

inhibitor U0126 and calcium chelating reagent BAPTA from Topscience Co., Ltd (Boston, USA), IP_3R inhibitor 2-APB and DIDS (Sigma-Aldrich), 5-FU and SN-38 from MCE were dissolved in DMSO to 10 mM stock solution and stored at -20°C. ER stress inhibitor tauroursodeoxycholate (HY-19696, MedChemExpress, USA) was dissolved in sterile ddH_2O to 20 mM stock solution and stored at -20°C.

MTT assay. Cells were seeded in 96-well plates at a density of 3×10^3 cells in 200 μL/well and treated with SR140333, Aprepitant, 5-FU or SN-38 at various concentrations or vehicle alone at different time points as indicated. Then 10 μL MTT (3- [4, 5-dimethyl-2-thiazolyl]-2, 5-diphenyl-2 H-tetrazolium bromide) solution (5 mg/mL, Sigma-Aldrich, Missouri, USA) was added to each well and incubated at 37°C in a humidified atmosphere with 5% CO_2 for 4 hours. The medium was then removed and 200 μL DMSO was added to dissolve the formazan crystals. The absorbance was measured at 570 nm using a microplate reader (Varioscan Flash, Thermo).

Plasmid construction. shRNA targeting human NK-1R was designed and chemically synthesized as NK-1R-shRNA (5'-GCCAGUAUCUACUCCAUGAUU-3'). The control vector was pGLV3-GFP-puro. The human c-Myc cDNA was designed and chemically synthesized as c-Myc cDNA. The control vector was pCDH-CMV-MCS-EF1-GFP-puro. Cells were transfected via lipofectamine 2000 (Invitrogen) and selected with 4 μg/mL puromycin (Solarbio Life Science, Beijing, China). The plasmids for human $HRas^{G12V}$ overexpression (pBabe-puro and pBabe-puro-$HRas^{G12V}$) were the generous gifts from Professor Rick Pearson in Peter MacCallum Cancer Centre, Australia.

Cell growth curve. Cells were seeded in 12-well plates at a density of 6×10^3 cells in 1 mL/well and harvested cells at indicated time point following treatment. The number of cells was measured by trypan blue exclusion assay (Count Star, Ruiyu Biotech Co., Ltd, Shanghai, China) everyday for 6 days.

Cell viability assay. Cells were seeded in 12-well plates at a density of 2×10^5 cells in 1 mL/well and pre-treated either the membrane-permeable calcium chelator BAPTA (10 μM), IP_3R inhibitor 2-APB (10 μM), anion exchange inhibitor DIDS (10 μM) or ER stress inhibitors tauroursodeoxycholate (10 μM) for one hour before adding SR140333 or Aprepitant. Cells were then h arvested and counted (Count Star, Ruiyu Biotech Co., Ltd, Shanghai, China).

Flow cytometric analysis of cell cycle. Cells were seeded in 6-well plates at a density of 5×10^5 cells in 3 mL/well and treated with SR140333 at various concentrations or vehicle alone for 24 hours. Cells were harvested and fixed in ice-cold ethanol for 24 hours at -20°C. After incubation in 500 μL cell cycle buffer containing 0.25 mg/mL DNase-free RNase at 37°C for 30 minutes, cells were stained with 5 μL PI (50 μg/mL, in 0.1% Triton X-100) for 30 minutes in dark according to the instruction of Cell Cycle Kit (Kaiji Bio Co., Nanjing, China) before analysis using FACS Aria (BD Biosciences, Mountain View, CA, USA). The fluorescence data was analyzed using the Novoexpress software and expressed as means ± SEM of three independent experiments.

Flow cytometric analysis of cell apoptosis. Cells were seeded in 6-well plates at a density of 5×10^5 cells in 3 mL/well and treated with SR140333 at various concentrations or vehicle alone for 24 hours. Cells were then harvested and incubated

in 500 μL binding buffer containing 5 μL Annexin V-FITC for 5 minutes followed by 5 μL PI for 30 minutes in dark according to the instruction of Cell Apoptosis Kit (Kaiji Bio Co., Nanjing, China) before analysis using BD Accuri C6 (BD Biosciences, Mountain View, CA, USA). The fluorescence data were analyzed using the FlowJo Software and expressed as means ± SEM of three independent experiments.

Protein extraction. To extract proteins from cells, after ice-cold PBS wash, cells were lysed in WSB lysis buffer containing 0.5 mM EDTA (ethylene diamine tetraacetic acid), 20 mM HEPES (2-[4-(2-Hydroxyethyl)-1- piperazinyl] ethanesulfonic acid), 2% SDS (Sodium Dodecyl Sulfonate) and 1 mM PMSF. To extract proteins from tumor tissues, snapped frozen tissue blocks were grinded in liquid nitrogen in mortars and then in homogenizer before solubilized in WSB lysis buffer.

Western blotting. 40 μg of lysates was loaded and run on a 8-12% SDS-PAGE gel and electrotransferred to PVDF membranes. The PVDF membranes were then blocked with 5% Albumin Bovine V (Roche, USA) in Tris-buffered saline which contained 0.1% Tween 20 at room temperature for 2 hours. The membranes were then incubated overnight with the specific primary antibodies of interesting proteins, including anti-Cyclin D1 (BS1741), CDK 4 (BS6462), p15 (BS1267), Cyclin B1 (BS1392), Caspase 9 (BS3188), Caspase 8 (AP0358), BAX (BS2538), ERK (BS1112), SP (BS1598), NK-1R(BS2632), GRP94 (BS90606), CHOP (K121) (BS1136), eIF2α (I45) (BS3651), phospho-eIF2α (S51) (BS4787) , ATF4 (BS1026),ATF6(BS6476), GRP78 (BS1154), GAPDH (AP0063) from Bioworld Technology, Inc, Nanjing, China; CDK 1 (SAB4500050), p21 (SAB4500065), BCL-2

(SAB4500003) and p-ERK (M9692) from Sigma-Aldrich, Missouri, USA; Cleaved Caspase 3 (ARG54938, Arigo biolaboratories Corp., Taiwan, China); PARP (#9532), p-ERK (Thr202/Tyr204) (#4370S,) from Cell Signal Technology Inc., MA, USA; c-Myc (ab32072, Abcam Biotechnology, Inc., Hong Kong, China); p-PERK(Thr982)(DF7576), PERK(AF5304), p-IRE1(Ser724)(AF7150), IRE1(DF7709) from Affinity Biosciences,OH, USA; XBP1(A1731), P-gp (A11758), ABCC1 (A11153), ABCG2 (A5661) from ABclonal Biotech Co., Ltd, Wuhan, China. Specific proteins were visualized by chemiluminescence with BiodlightTM ECL Chemiluminescent HRP Substrate (Bioworld Technology, Inc, Nanjing, China) using a Tanon 5500 Chemiluminescence Detection System (Tanon, Shanghai, China).

Calcium analysis. Cells were seeded in cover glass-bottom dishes (SPL, Korea) designed for confocal microscopic examination at a density of 2×10^5 cells in 1 mL/dish and cultured for 24 hours. The cells were rinsed three times with the assay buffer (130 mM NaCl, 5 mM KCl, 10 mM HEPES, 8 mM D-glucose, 1.2 mM $MgCl_2$, and 1.5 mM $CaCl_2$, pH 7.4) and then incubated with 1 mL assay buffer containing an organic anion transport inhibitor probenecid (2.5 mM, Sigma-Aldrich, Missouri, USA) and 0.1% Pluronic F-127 (Invitrogen, San Diego, CA, USA) plus either 1 μM Fluo-4 AM (Invitrogen, San Diego, CA, USA) or 2 μM Rhod-2 AM (Invitrogen, San Diego, CA, USA), for 60 minutes at 37°C in a humidified atmosphere with 5% CO_2. After rinsing cells three times with assay buffer, the intracellular Ca^{2+} concentration was measured at 488 nm for Fluor-4 AM or 525 nm for Rhod-2 AM using a laser scanning confocal microscope (CISI, Nikon, Tokyo, Japan). After the initial measurement for 45 seconds to determine the baseline fluorescence, SR140333 at 20, 40 and 60 μM for HCT116 cells, 10, 20 and 40 μM for SW620 cells, or SP at 500 nM and 10 μM, was added. Image recording continued for 1500 seconds after stimulation

of SR140333 and for 3000 seconds after stimulation of SP. Ca^{2+} concentrations were expressed as the average fluorescence intensity of 20 cells/field randomly selected from at least three fields at each time point. The fluorescence data were analyzed using the EZ-C13.20 Free Viewer software (Nikon, Tokyo, Japan) and expressed as means ± SEM of three independent experiments.

Determination of intracellular reactive oxygen species (ROS). The intracellular ROS were measured by flow cytometry using a cell-based ROS assay kit (S0033, Beyotime Biotechnology, Beijing, China) and MitoSOX (M36008, Invitrogen, USA) following the manufacturer's instruction. Cells were seeded in 6-well plates at a density of 5×10^5 cells in 3 mL/well and treated with SR140333 at 38 μM or 43 μM for indicated time points. Cells were then incubated with 10 μM of cell-permeable 2',7'-dichlorofluorescein diacetate (DCFH-DA) or 5 μM MitoSOX, for 20 min at 37 ℃ before harvest. After washing cells with medium (FBS free) three times, the fluorescence was quantitatively measured at an excitation: emission of 488 nm : 525nm by flow cytometry (BD Accuri C6, BD Biosciences, Mountain View, CA, USA).

Xenograft growth experiments. Female BALB/c nude mice of 4 weeks old were obtained from SLAC company (Shanghai, China). To develop the tumor mouse model, HCT116 cells were harvested and washed with PBS and administrated subcutaneous injected into the flanks at a density of 5×10^6 cells in 200 μL normal saline per mouse. When tumors reached 100–150 mm^3, the mice were divided randomly into two groups (n = 6 mice for SR140333 group and n = 5 mice for vehicle group). For the treatment group, mice were treated with SR140333 at a dose of 10 mg/kg via peri-tumoral injection once daily for 16 days. For the vehicle group, the

mice were treated with vehicle via *in situ* injection. Tumor size was measured by vernier caliper every day. Tumor volume (mm^3) was calculated using the following formula: V ($mm^{3)}$ = 1/2×a×b^2, where a and b were the longest and widest diameter of tumor, respectively. At the end of the experiment, all animals were sacrificed according to institutional guidelines. Tumors, brains, viscera including hearts, livers, spleens, lungs and kidneys of nude mice in both SR140333 group and vehicle group were resected, some of which were fixed in formalin for paraffin embedding and others were stored at-80°C for Western Blotting. All experimental protocols were performed in accordance with instruction guidelines from the China Council on Animal Care and approved by the guidelines of the Ethics Committee of Animal Experiments at Zhejiang Sci-Tech University (NO.20200409-4).

Female BALB/c nude mice aged 4-6 weeks were purchased and placed in the Animal Experiment Center of Hangzhou Normal University. All operations are carried out in accordance with Hangzhou Normal University's laboratory animal care and use standards. HCT116/5-FU cells (about 5×10^6 cells) were injected subcutaneously into the right back of each mouse. After cell inoculation, when the tumor formed reached 100-150 mm^3, the animals were randomly divided into 6 groups (n= 5 mice in each group). The first group of mice received intra-tumoral injection of dissolved coal (10% corn oil and 5% DMSO) as a control. The second to fourth group received 5-FU (10 mg / kg) or SR140333 (10 mg / kg) or Aprepitant (10 mg/kg) treatment, group 5 received 5-FU (10 mg / kg) and SR140333 (10 mg / kg) treatment, and group 6 received 5-FU (10 mg / kg) and Aprepitant (10 mg / kg) treatment. Peri-tumoral injection every 2 days for 18 days. The tumor size was measured using a vernier caliper every two days, and the tumor volume was calculated as V (mm^3) =1/2×a×b^2

28 days after tumor cell inoculation, the experiment was terminated and the mice were sacrificed. The tumor and organs of each mouse were excised and their weight was measured.

H&E-staining. Resected tumors were immersed in 4% paraformaldehyde overnight in 12-well plates and then transferred to increasing concentrations of ethanol to dehydration, which were followed by paraffin embedding and section. Before staining, 7-μm-thick sections were dewaxed in xylene, rehydrated through decreasing concentrations of ethanol, and washed with PBS. And then tumors were stained with hematoxylin and eosin for 2 minutes and 3 minutes respectively. After staining, sections were dehydrated through increasing concentrations of ethanol and xylene, mounted and photographed (Olympus BX53, Olympus, Japan).

TUNEL staining. Resected tumors were immersed in 4% paraformaldehyde overnight in 12-well plates and then transferred to increasing concentrations of ethanol to dehydration, which were followed by paraffin embedding and section. Before staining, 5-μm-thick sections were dewaxed in xylene, rehydrated through decreasing concentrations of ethanol, and washed in PBS. TUNEL Apoptosis Detection Kit (Alexa Fluor 488) (Roche, USA) was applied for TUNEL staining. Experiment procedure was performed according to the manuscript instruction. 4,6-Diamidino-2-phenylindole was used to stain the nuclei and then photographed (ZEISS Axio Vert A1, ZEISS, Germany).

Patients and tissue samples. A total of 50 colon cancer patients received colectomy from 2012 to 2015 in the 903rd Hospital of PLA, Hangzhou, China, were included in the present study. Sufficient tissue samples and complete clinic pathological

information for each patient were collected. The paired cancerous and non-cancerous tissues from these patients were routinely fixed in 10% buffered formalin and blocked in paraffin, ready to immunohistochemistry assay. All patients had not received any anticancer therapy before surgery, and they were followed up every 6 months until death or study end (September 30, 2017). The tissue samples were obtained with patient informed consent, and the protocol was approved by Institutional Review Board of the 903rd Hospital of PLA (NO.20140512/03/01/005).

Immunohistochemistry. For each of 50 colon cancer patients, paraffin-embedded cancerous and matched non-cancerous samples were cut into consecutive 4 μm sections. Immunohistochemistry assay for NK-1R expression was performed using an UltraSensitive™ SP kit (KIT-9710, Maixin, Fuzhou, China) according to the manufacturer's instructions. Briefly, the sections were deparaffinized in xylene, rehydrated with graded ethanol, and subjected to antigen retrieval in citrate buffer (pH 6.0) in a high-pressure cooker. The sections were subsequently blocked for endogenous peroxide activity with 3% hydrogen peroxide, treated with preimmune goat serum to block nonspecific binding sites, and then incubated with the primary rabbit polyclonal antibody against human NK-1R (Novus, NB300-119SS; dilution 1:200) at 4°C overnight. The sections were washed and incubated with a secondary biotinylated anti-mouse/rabbit antibody. The immunostaining was visualized with a diaminobenzidine detection kit (DAB-0031, Maixin, Fuzhou, China) and then the sections were counterstained with hematoxylin, dehydrated, cleared, and coverslipped.

Immediately after harvest, tumors were fixed in 4% PFA for 24 h and paraffin-embedded. with anti-p-ERK1/2 (Cell Signal Technology, #4370S, dilution

1:100), c-Myc (Abcam Biotechnology, ab32072, 1:400), ABCC1(ABclonal Biotech Co, A11153, 1:400), ABCG2(ABclonal Biotech Co, A5661, 1:400), p-PERK (Affinity Biosciences, DF7576, 1: 100), ATF4 (Bioworld Technology, BS1026, 1:400), CHOP(K121) (Bioworld Technology, BS1136, 1:400) antibody for immunohistochemical staining.

Evaluation of immunostaining and statistical analysis. Brown cytoplasmic staining in the colon cancer cells or non-cancerous epitheliums was defined as positive staining. The signal was quantified by the Allred score system which represented the estimated proportion of positive staining cells combined with staining intensity, as previously described. In brief, the proportion score represented the estimated proportion of positive tumor cells (0, none; 1, <1/100; 2, 1/100 to 1/10; 3, 1/10 to 1/3; 4, 1/3 to 2/3; and 5, >2/3), and the intensity score represented the average intensity of positive tumor cells (0, none; 1, weak; 2, intermediate; and 3, strong). The proportion and intensity scores were then added to obtain a final score, which ranged from 0 to 8. A score ≥ 3 was designated as positive expression and a score of 0 or 2 was regarded as negative. The stained sections were viewed by two individuals independently using an Olympus CX31 microscope (Olympus, Japan). The associations between NK-1R immunostaining and clinic pathological factors were determined using Chi-square test. The probability of survival was estimated by Kaplan-Meier method and compared by log-rank test. All *P* values were two-sided and less than 0.05 was considered significant. Statistical analyses were performed by the SPSS 17.0 for windows (SPSS, Chicago, IL, USA).

Supplemental Figures

Figure S1

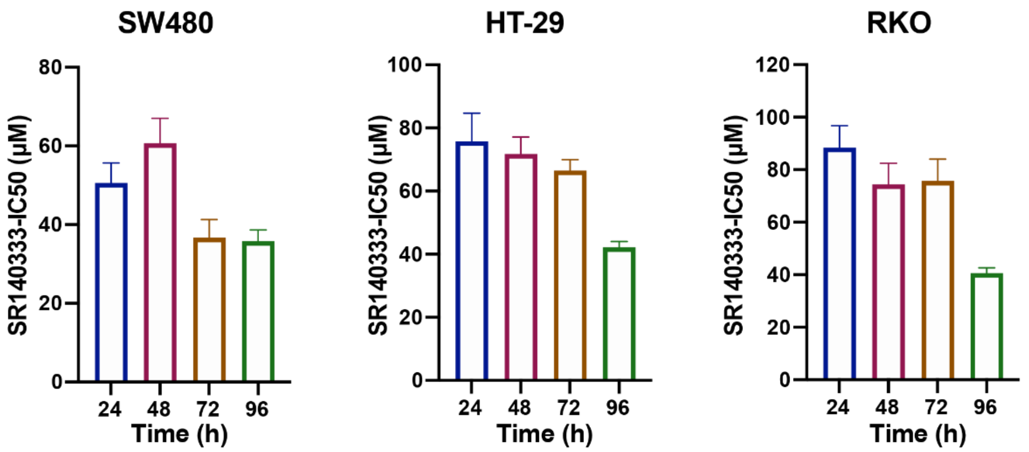

Figure S1 Blocking NK-1R by SR140333 inhibited cell proliferation in human colon cancer cells. IC50 values of SR140333 in different cell lines measured by MTT assay. Data are expressed as mean ± SEM, n = 3.

Figure S2

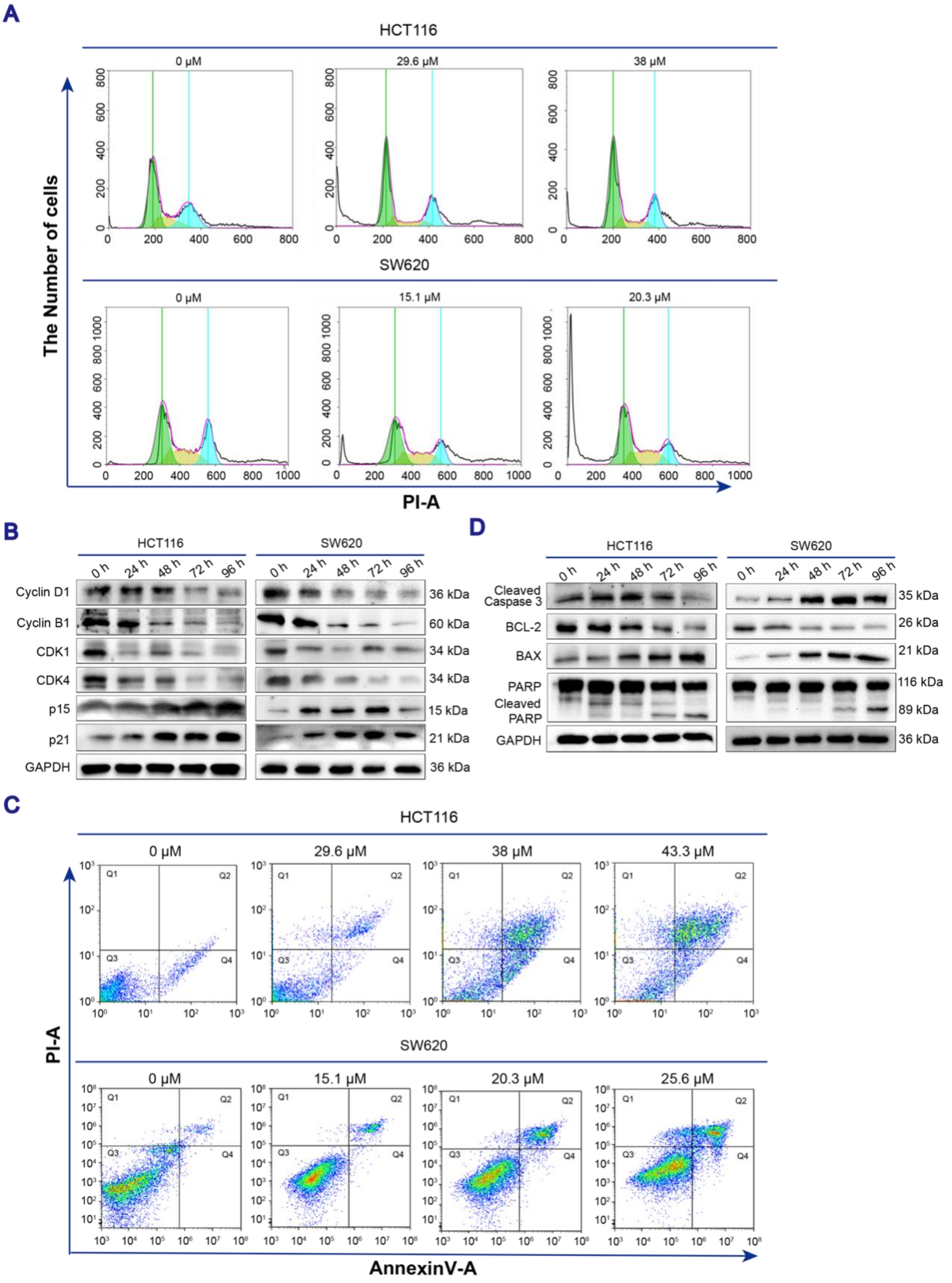

Figure S2 Blocking NK-1R induced cell cycle arrest and apoptosis in human colon cancer cells. (A) Cell cycle analysis of HCT116 and SW620 cells treated with SR140333 at the indicated doses for 24 hours by PI-binding assay. At least three independent experiments were performed and the representative results were shown.

(B) Western blotting of cell cycle-related proteins in HCT116 and SW620 cells treated with SR140333 at 38 μM and 20.3 μM, respectively, at the indicated time points. GAPDH was used as a loading control. At least three independent experiments were performed and the representative results were shown. (C) Annexin V plus PI analysis of HCT116 and SW620 cells treated with SR140333 at the indicated doses for 24 hours. At least three independent experiments were performed and the analysis of one representative experiment showed the proportion of early apoptotic cells (Q4 area), later apoptotic cells (Q2 area) and normal cells (Q3 area). (D) Western blotting of apoptosis-related proteins in HCT116 and SW620 cells treated with SR140333 at 38 μM and 20.3 μM, respectively, at the indicated time points. GAPDH was used as a loading control. At least three independent experiments were performed and the representative results were shown.

Figure S3

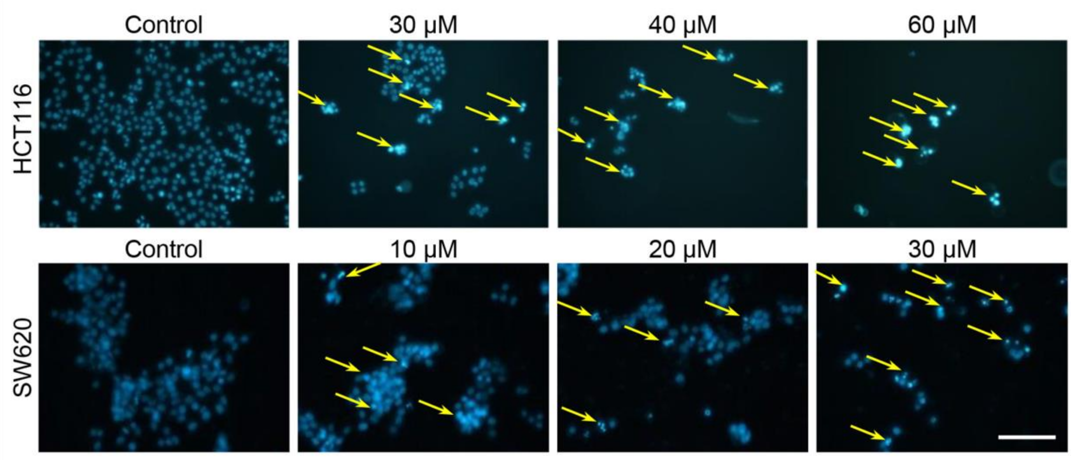

Figure S3 Blocking NK-1R induced apoptosis in human colon cancer cells. Representative images from three indepedent experiments of HCT116 and SW620 cells treated with SR140333 at indicated doses for 24 hours and stained with Hoechst33342. Scale bar, 50 μm.

Figure S4

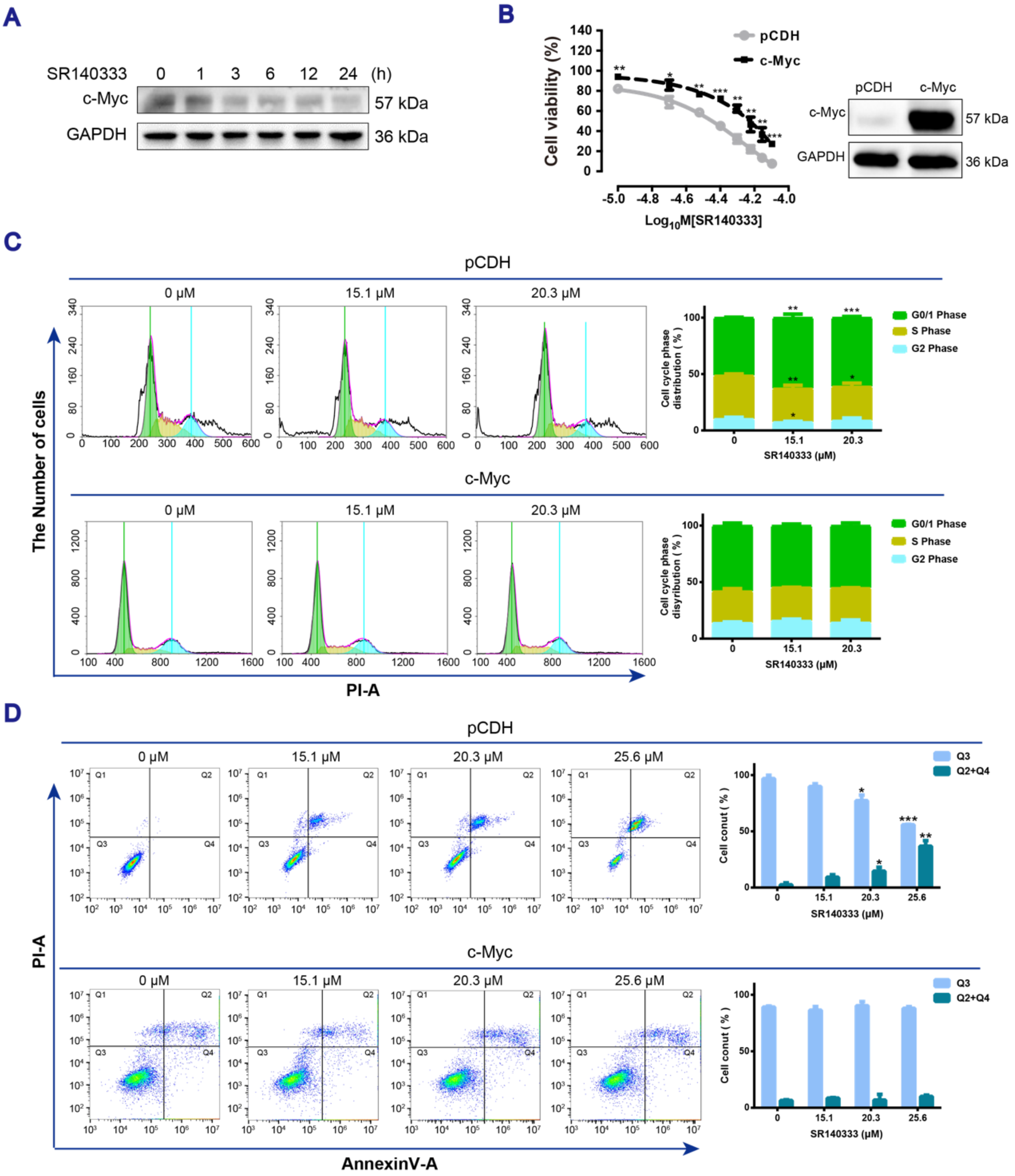

Figure S4 Decrease of c-Myc mediates cytotoxicity of SR140333 in human colon cancer cells. (A) Western blotting of c-Myc expression in SW620 cells treated with 20.3 µM SR140333 at the indicated time points. Representative images from at least three independent experiments are shown. (B) SW620 cells were stably transfected with either empty vector pCDH or the vector expressing human c-Myc and treated with SR140333 at the indicted doses for 24 hours. Cell viability was measured by trypan blue exclusion assay. Data are expressed as mean ± SEM, n = 3. *P<0.05

**P<0.01 and ***P<0.001 by student's *t* test as compared with the cells transfected with empty vector. Western blotting of c-Myc expression. GAPDH was used as a loading control, representative images from at least three independent experiments are shown. (C) and (D) Cell cycle analysis and cell apoptotic analysis of SW620 cells with or without c-Myc overexpression treated with SR140333 at the indicated doses for 24 hours. Data presented as mean ± SEM, n = 3, P-values are calculated using one-way ANOVA with Dunnett correction, *P<0.05, **P<0.01, ***P<0.001,

Figure S5

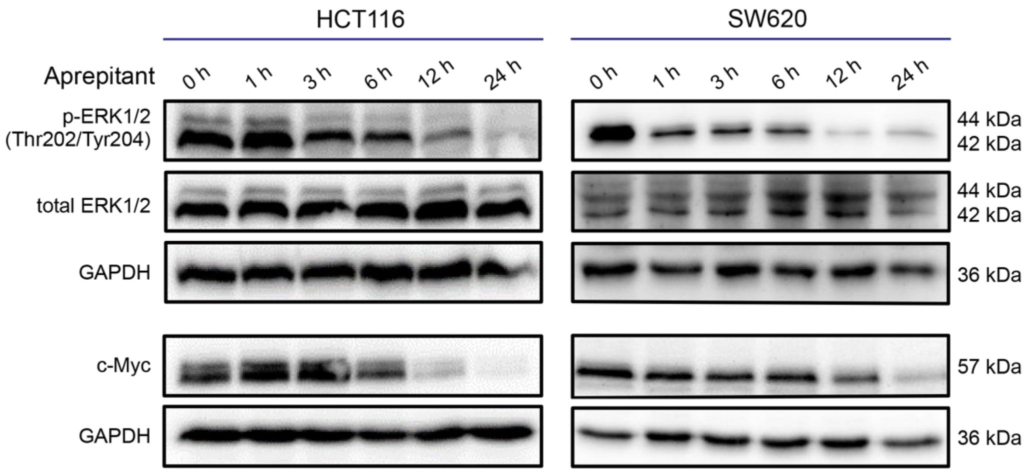

Figure S5 Aprepitant suppresses p-ERK1/2-c-Myc signaling pathway. Western blotting of p-ERK1/2, total ERK1/2 and c-Myc in HCT116 and SW620 cells treated with 40 μM Aprepitant at the indicated time points. GAPDH was used as a loading control. Representative images from at least three independent experiments are shown.

Figure S6

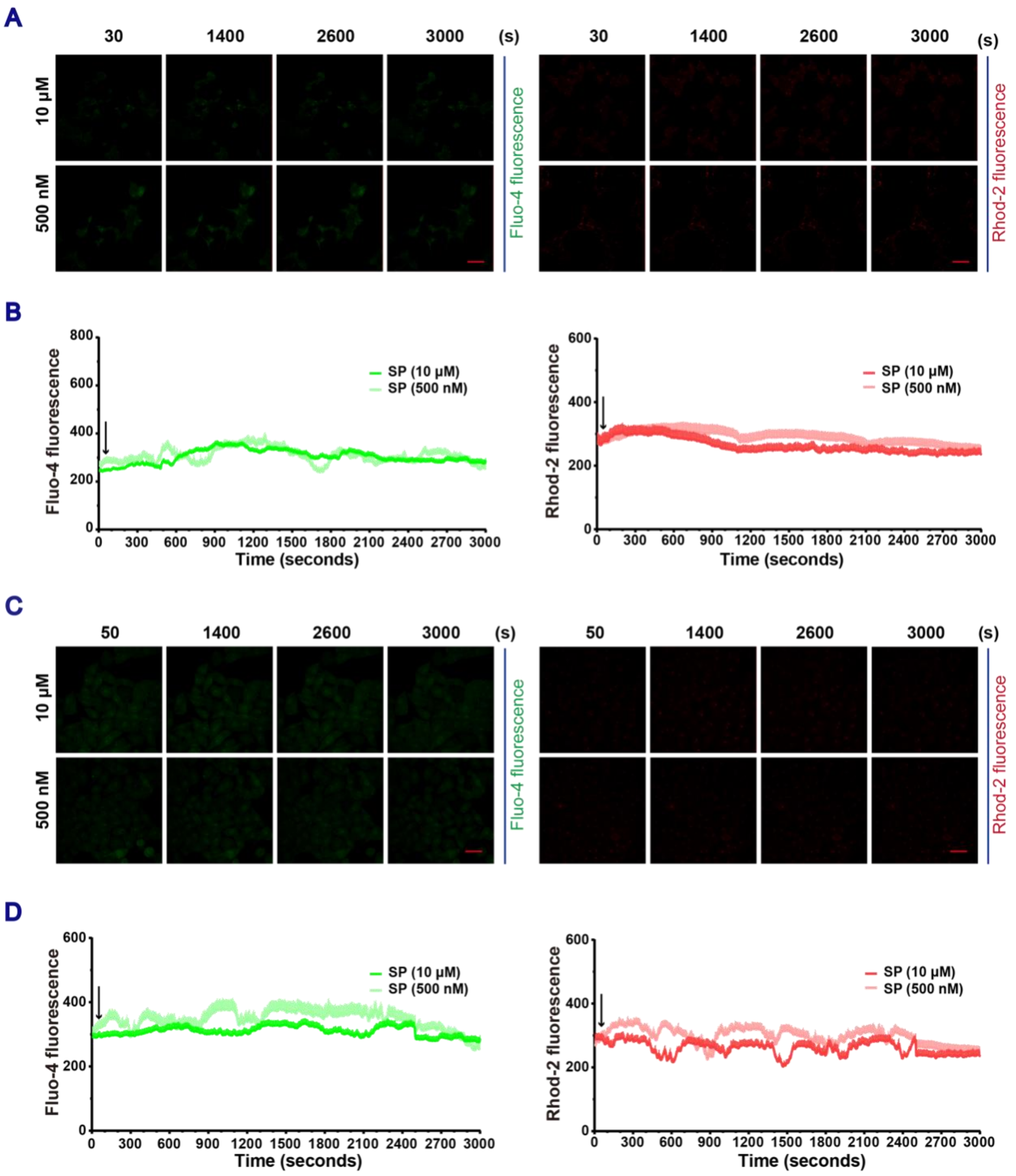

Figure S6 Stimulation of NK-1R with SP in HCT116 and SW620 cells did not affect cytosolic and mitochondrial calcium flux. Images of intracellular fluorogenic calcium indicator Fluo-4 AM staining and mitochondrial fluorogenic calcium indicator Rhod-2 AM staining in (A) HCT116 cells and (C) SW620 cells before and after treatment with SP. Scale bar, 50 μm. The mean fluorescence intensity of 20 cells ((B) HCT116 cells and (D) SW620 cells) randomly picked up from at least two fields were calculated and presented as means ± SEM from at least three independent

experiments. Arrow indicated the time of adding SP after initial measurement for 50 seconds.

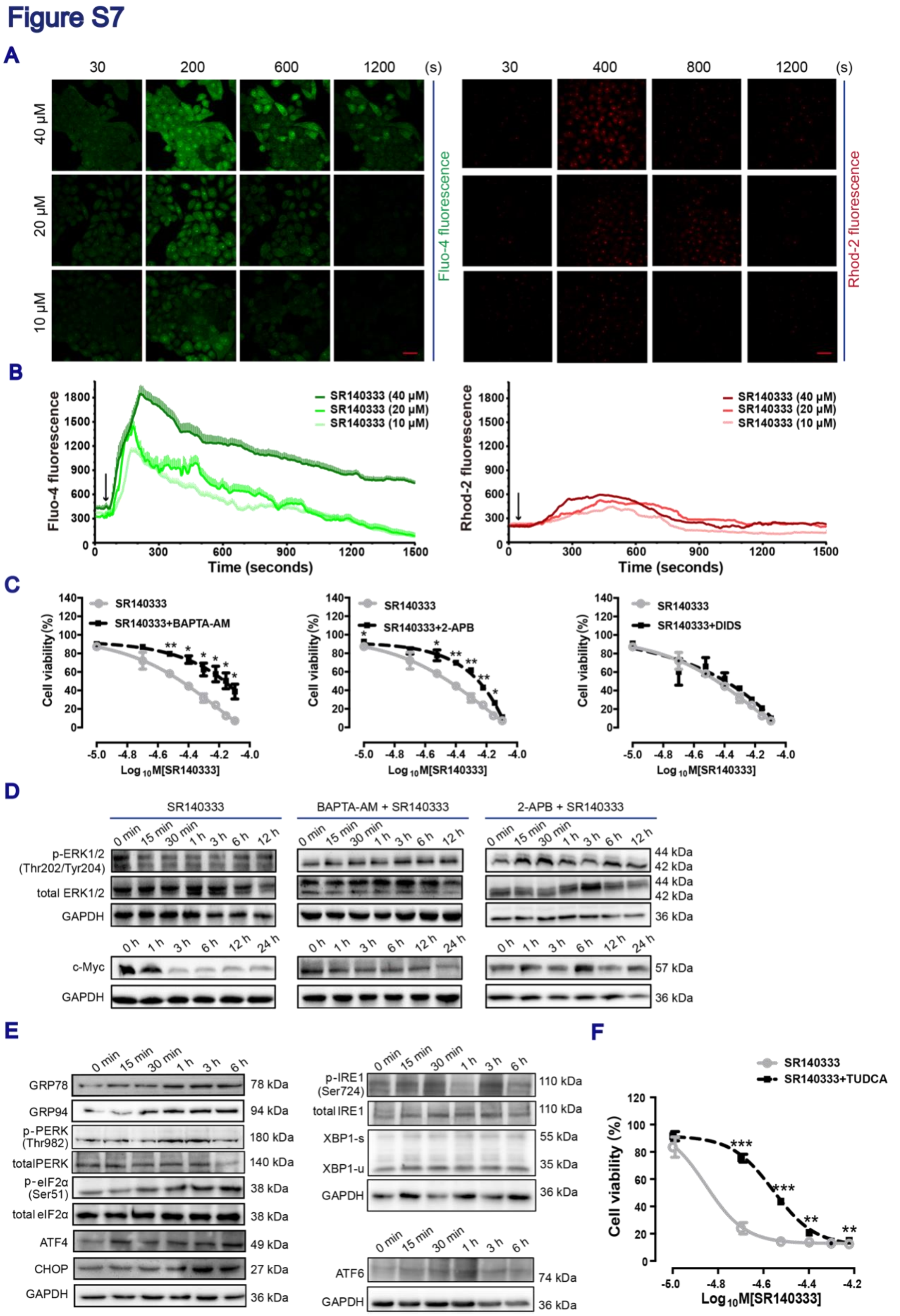

Figure S7 Blocking NK-1R induces ER calcium release and ER stress in SW620 cells. (A) Images of intracellular fluorogenic calcium indicator Fluo-4-AM staining and mitochondrial fluorogenic calcium indicator Rhod-2-AM staining in SW620 cells

before and after treatment with SR140333 at 10, 20 and 40 μM. Scale bar, 50 μm. (B) The mean fluorescence intensity of 20 cells randomly picked up from at least two fields were calculated and presented as means ± SEM from at least three independent experiments. Arrow indicated the time to add SR140333 after initial measurement for 50 seconds. (C) SW620 cells were pre-treated with either BAPTA-AM (10 μM), 2-APB (10 μM) or DIDS (10 μM), followed by SR140333 administration at the indicated concentrations for 24 hours. Cell viability was measured by trypan blue exclusion assay. Data presented as mean ± SEM, n = 3, P-values are calculated using tudent's *t* test, $*P<0.05$ and $**P<0.01$, compared with the group treated with SR140333 single agent alone. (D) Western blotting of p-ERK1/2 and c-Myc expression in SW620 cells treated with SR140333 (20.3 μM) at the indicated time points after pre-treatment with BAPTA-AM (10 μM) or 2-APB (10 μM) for 1 hour. Representative images of at least three independent experiments were shown. (E) Western blotting of ER stress markers in SW620 cells treated with SR140333 at 20.3 μM. GAPDH was used as a loading control. All the Western blotting images are representative of at least three independent experiments. (F) The viability of SW620 cells treated with SR140333 single agent or in combination with the ER stress inhibitor TUDCA (10 μM) for 24 hours as measured by MTT. Date presented as mean± SEM, n = 3, $**P<0.01$, and $***P<0.001$ by student's *t* test as compared with the group treated with SR140333 single agent alone.

Figure S8

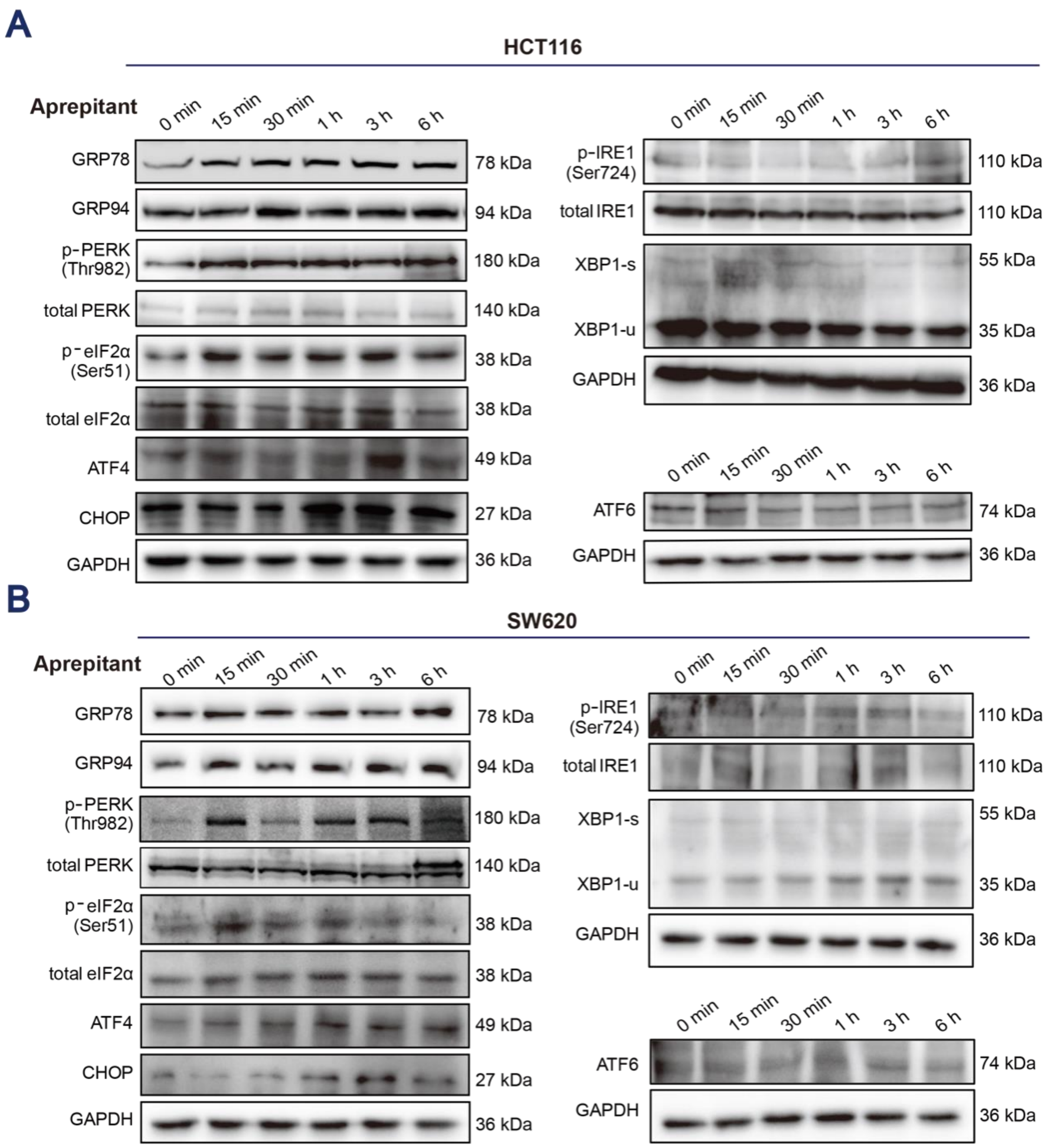

Figure S8 Aprepitant induces ER stress. Western blotting of ER stress markers in HCT116 cells (A) and SW620 cells (B) treated with Aprepitant at 40 μM. GAPDH was used as a loading control. All the Western blotting images are representative of at least three independent experiments.

Figure S9

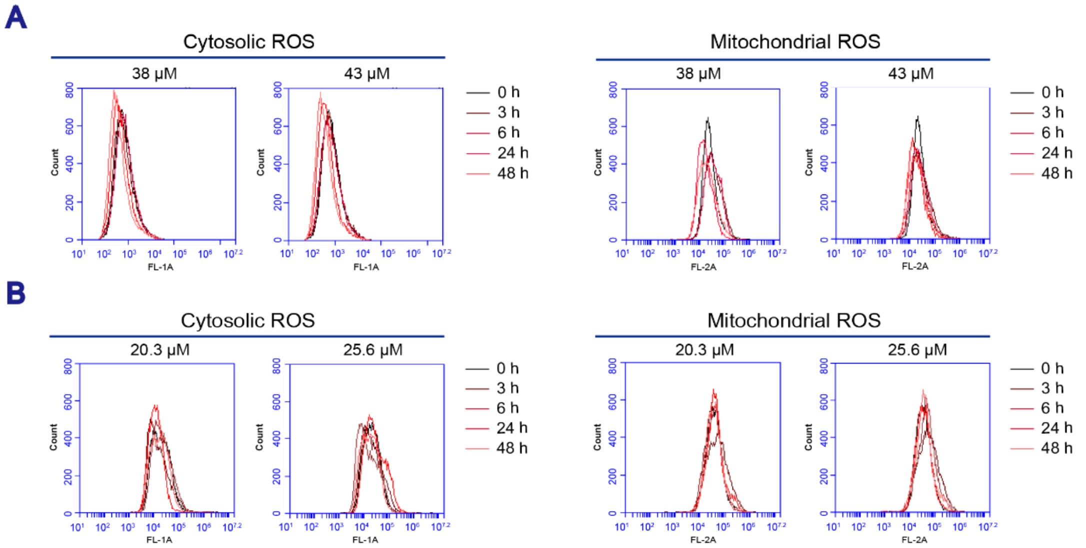

Figure S9 SR140333 did not affect reactive oxygen species production in HCT116 and SW620 cells. (A) and (B) The cytosolic and mitochondrial superoxide levels in HCT116 cells (A) and SW620 cells (B) were measured by cell-permeable 2',7'-dichlorofluorescein diacetate (DCFH-DA) at 10 μM and MitoSOX at 5 μM, respectively. Representative images of at least three independent experiments were shown.

Figure S10

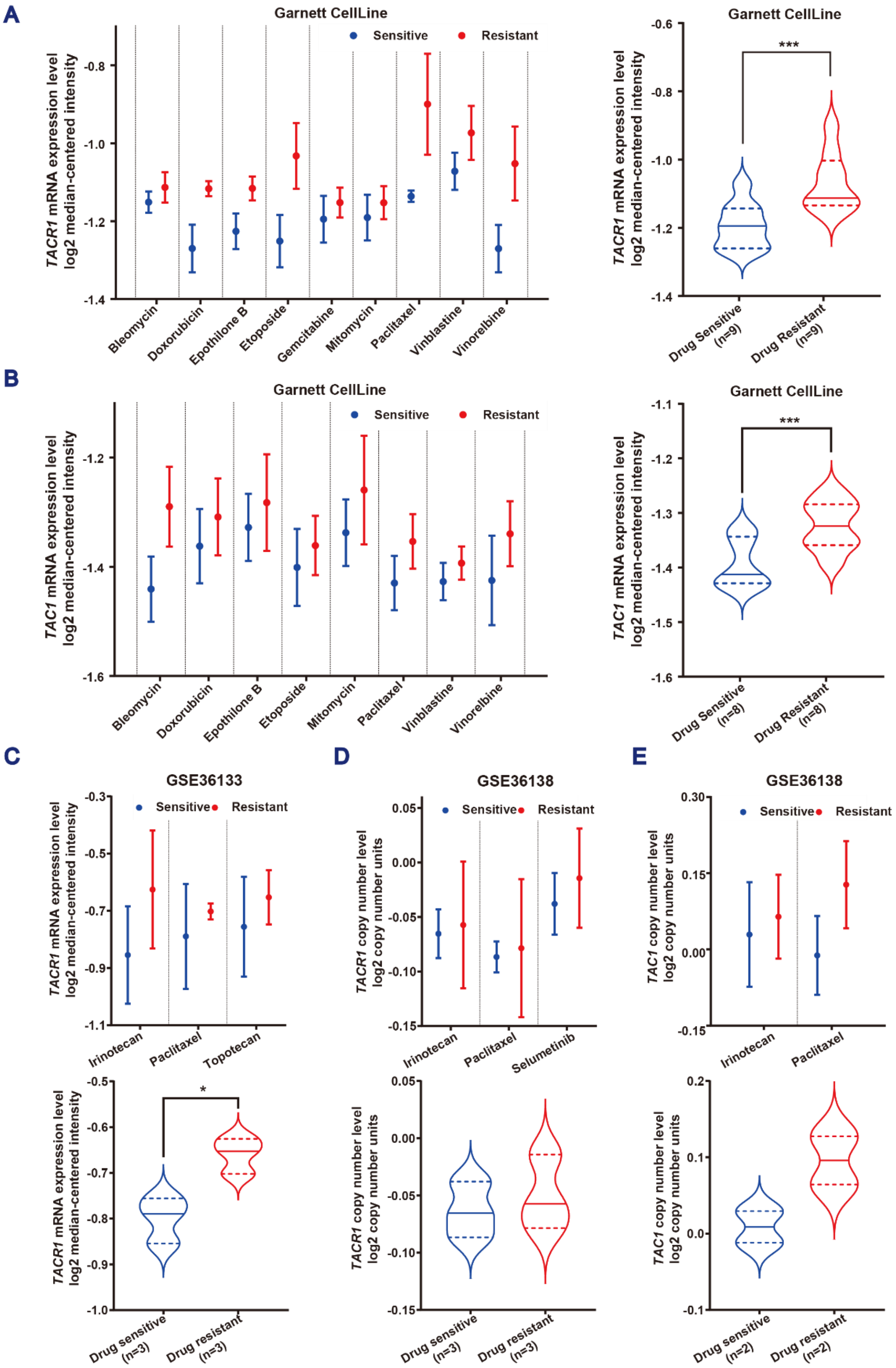
A
Garnett CellLine
Sensitive
Resistant
TACR1 mRNA expression level
log2 median-centered intensity
Bleomycin
Doxorubicin
Epothilone B
Etoposide
Gemcitabine
Mitomycin
Paclitaxel
Vinblastine
Vinorelbine
***
Drug Sensitive (n=9)
Drug Resistant (n=9)
B
TAC1 mRNA expression level
Drug Sensitive (n=8)
Drug Resistant (n=8)
C
GSE36133
Irinotecan
Topotecan
Drug sensitive (n=3)
Drug resistant (n=3)
*
D
GSE36138
TACR1 copy number level
log2 copy number units
Selumetinib
E
TAC1 copy number level
Drug sensitive (n=2)
Drug resistant (n=2)

Figure S10 The mRNA expression and copy number levels of TACR1 and TAC1 in drug-sensitive and drug-resistant CRC cell lines in the Oncomine database. (A) and (B) The mRNA expression levels of *TACR1* (A) and *TAC1* (B) in drug-sensitive and drug-resistant CRC cell lines in the dataset "Garnett Cell line" extracted from the Oncomine database (www.oncomine.org). Data are expressed as means ± SEM, ***P<0.001 by student's t test. (C) The mRNA expression levels of *TACR1* in drug-sensitive and drug-resistant CRC cell lines in the dataset "GSE36138" extracted from the Oncomine database. Data are expressed as means ± SEM. *P<0.05 by student's t test. (D) and (E) The copy number levels of *TACR1* (D) and *TAC1* (E) in drug-sensitive and drug-resistant CRC cell lines in the dataset "GSE36138" extracted from the Oncomine database.Data are expressed as means ± SEM. *P<0.05 by student's t test .

Figure S11

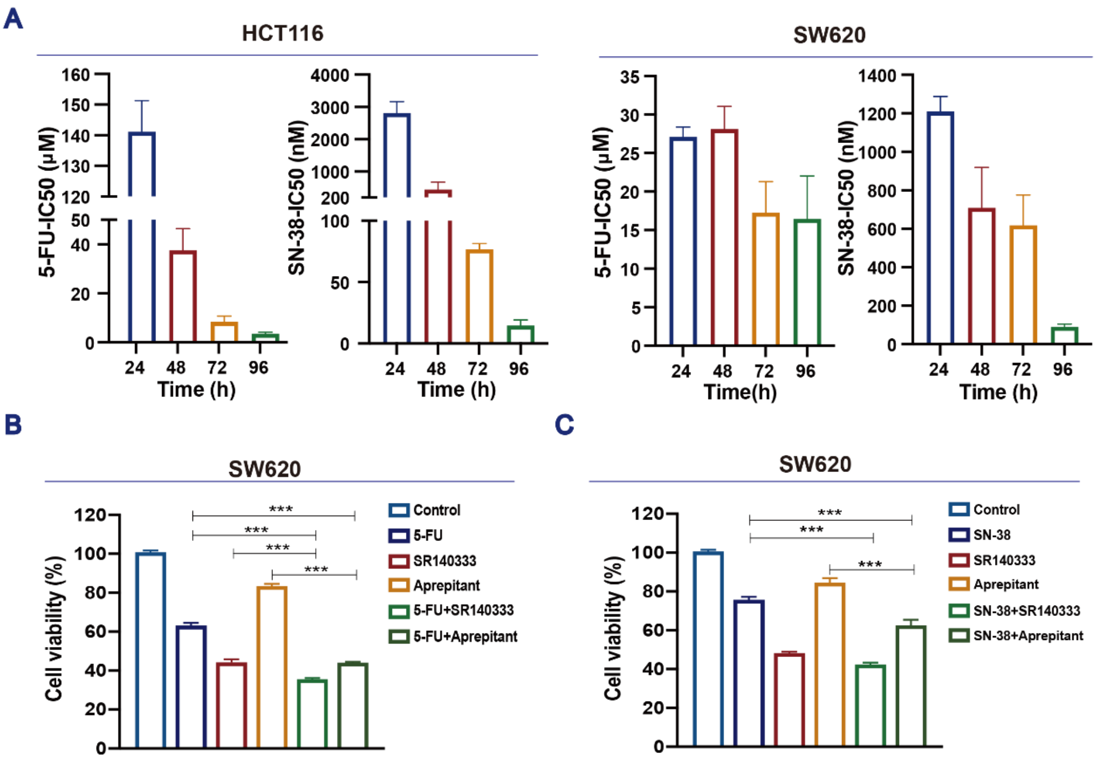

Figure S11 Blocking NK-1R enhances the sensitivity of CRC cells to chemotherapeutic drugs. (A) The IC50 of 5-FU and SN-38 in HCT116 cells and SW620 cells measured by MTT assay. Values represent means ± SEM (n = 3). (B) Cell viability upon treatment with 5-FU (60 μM), SR140333 (15 μM) or Aprepitant (30 μM) for 24 hours in SW620 cells. Data presented as mean ± SEM, n=3, P-values are calculated using one-way ANOVA with Tukey correction, ****P*<0.001.(C) Cell proliferative viability of combination SN-38 (300 nM) with SR140333 (15 μM) or Aprepitant (30 μM) at 24 hours in SW620 cells. Data presented as mean ± SEM, n=3, P-values are calculated using one-way ANOVA with Tukey correction, ****P*<0.001.

Figure S12

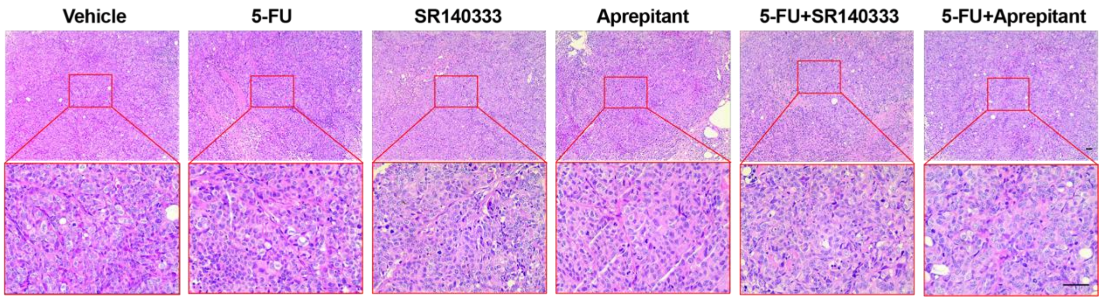

Figure S12 H&E staining of the tumor in mice treated with vehicle or Chemotherapy drugs. At least three independent experiments were performed and the representative results were shown. Scale bar: 50 μm.

Figure S13

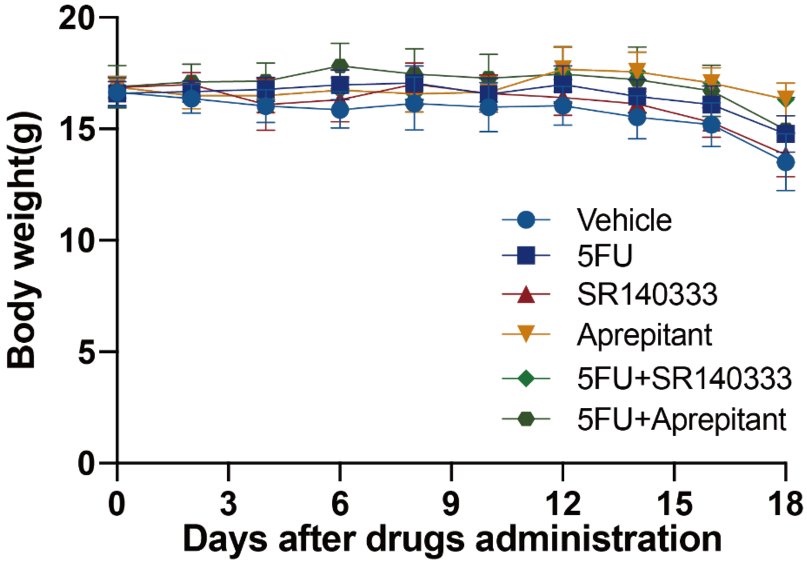

Figure S13 The body weights of mice with HCT116/5-FU xenografts following treatment. Mice were treated with 5-FU (10 mg/kg), SR140333 (10 mg/kg) or Aprepitant (10 mg/kg) either as single agents or in combination every two days**.** Body weights of mice measured at the indicated time points. Date presented as means ± SEM, n = 5.

Figure S14

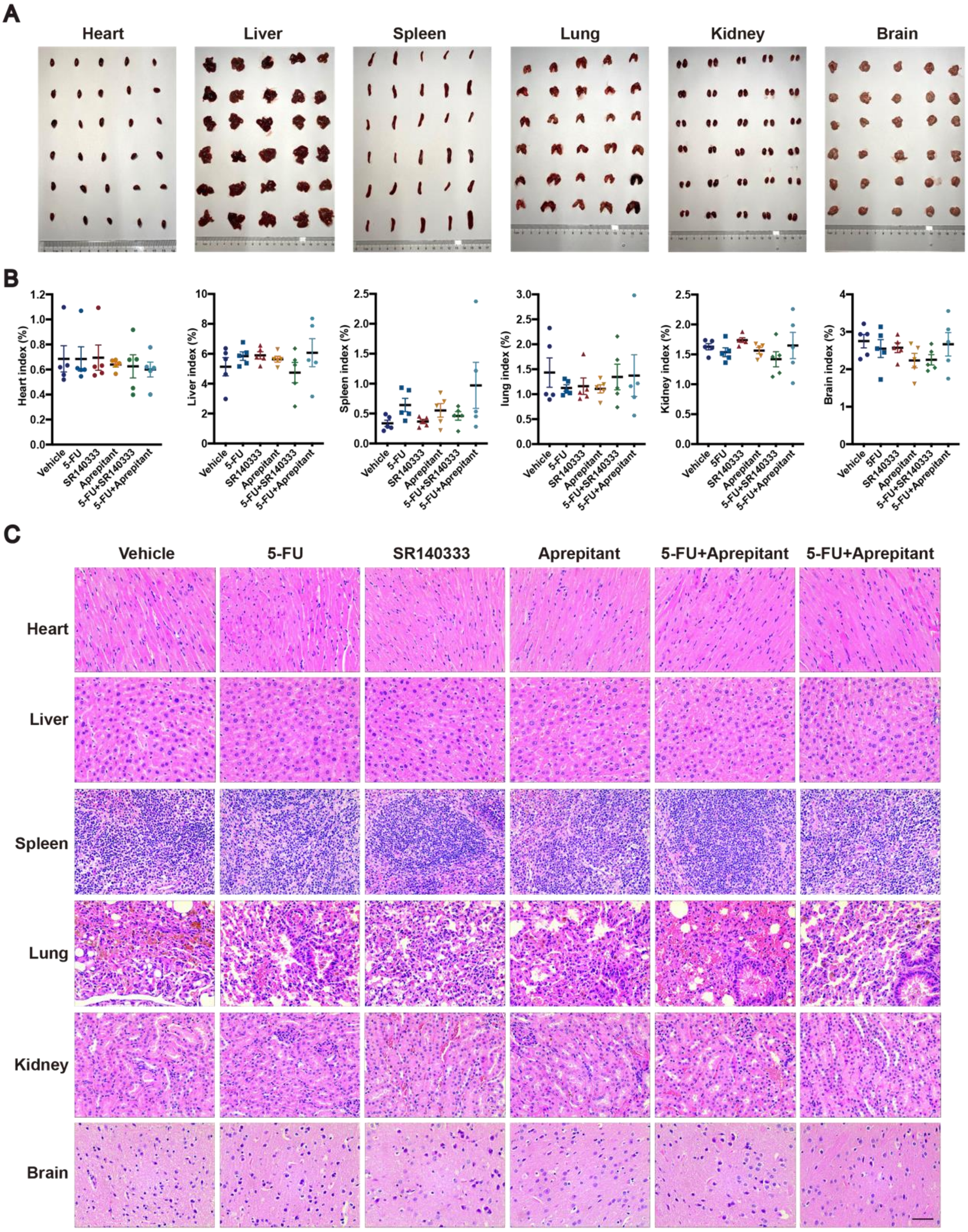

Figure S14 Chemotherapy drugs have no obvious toxic effects on mice. (A) The images of organs excised from all nude mice on day 18 (n = 5). (B) Organ weight index compared with vehicle group. Data are expressed as means ± SEM, n = 5. (C) H&E-staining of the heart, liver, spleen, lung, kidney and brain in mice treated with vehicle or Chemotherapy drugs. Scale bar: 50 μm.

Figure S15

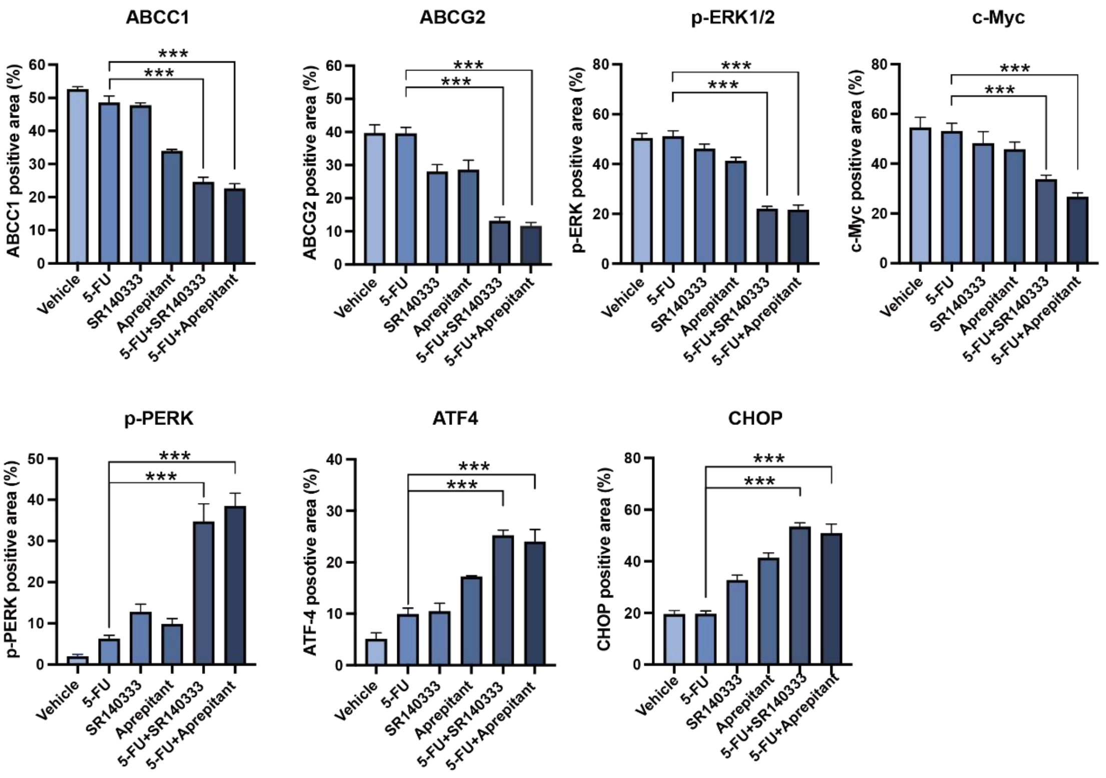

Figure S15 Quantification of positive immunohistochemistry staining in the tissue samples from HCT116/5-FU xenografts. IHC staining for drug-resistant proteins (ABCC1 and ABCG2), p-ERK1/2, c-Myc and ER stress-related proteins (p-PERK, ATF4 and CHOP) shown in Figure 7(E) were quantified as the percentage positive area. Data presented as mean ± SEM, n=4, P-values are calculated using one-way ANOVA with Tukey correction, ****P*<0.001.

Table S1 The information of colon cancer patients.

Patient	Age	Sex	Tumor site	TNM	Tumor stage	Outcome	Followup (month)
**P1**	68	male	Right-sided	T3 N2a	III	death	12
**P2**	46	male	Right-sided	T2 N0	I	survival	66
**P3**	61	male	Left-sided	T2 N0	I	survival	64
**P4**	66	male	Left-sided	T2 N1a	III	survival	64
**P5**	72	female	Left-sided	T3 N0	I	death	12
**P6**	54	female	Left-sided	T2 N1a	III	survival	63
**P7**	65	female	Right-sided	T3 N0	I	survival	63
**P8**	60	female	Right-sided	T2 N0	I	survival	62
**P9**	65	female	Right-sided	T1 N0	I	death	18
**P10**	71	male	Left-sided	T2 N0	I	survival	61
**P11**	67	female	Left-sided	T3 N0	I	survival	60
**P12**	75	male	Left-sided	T4 N0	I	death	12
**P13**	38	female	Left-sided	T4 N2b	III	death	17
**P14**	72	female	Right-sided	T3 N0	I	death	58
**P15**	77	male	Left-sided	T2 N0	I	survival	58
**P16**	59	male	Right-sided	T4 N2a	III	death	16
**P17**	67	female	Right-sided	T2 N1a	III	survival	57
**P18**	65	female	Left-sided	T4 N0	I	death	56
**P19**	74	female	Left-sided	T2 N0	I	death	54
**P20**	50	female	Left-sided	T3 N0	I	death	24
**P21**	62	female	Left-sided	T2 N0	I	survival	53
**P22**	53	male	Right-sided	T3 N0	I	death	42
**P23**	51	female	Left-sided	T3 N2b	III	death	7
**P24**	48	male	Left-sided	T4 N2a	III	death	30
**P25**	74	female	Right-sided	T4 N2a	III	death	3
**P26**	53	male	Right-sided	T2 N0	I	survival	50
**P27**	73	female	Left-sided	T2 N0	I	survival	48
**P28**	71	male	Left-sided	T2 N0	I	death	47
**P29**	63	female	Right-sided	T2 N0	I	survival	47
**P30**	75	female	Right-sided	T2 N1a	III	survival	46
**P31**	78	male	Left-sided	T2 N0	I	death	46
**P32**	57	male	Right-sided	T2 N0	I	death	46
**P33**	57	male	Right-sided	T3 N1a	III	survival	45
**P34**	75	male	Left-sided	T2 N0	I	survival	44

**P35**	70	female	Right-sided	T3 N1a	III	death	24
**P36**	49	female	Right-sided	T2 N2a	III	survival	41
**P37**	74	female	Left-sided	T4 N0	I	death	5
**P38**	58	male	Left-sided	T2 N0	I	survival	40
**P39**	71	female	Right-sided	T3 N1a	III	survival	40
**P40**	71	male	Left-sided	T2 N0	I	survival	40
**P41**	71	male	Right-sided	T4 N2a	III	death	39
**P42**	48	male	Left-sided	T4 N0	I	death	39
**P43**	67	male	Right-sided	T3 N1a	III	death	5
**P44**	71	male	Right-sided	T3 N2b	III	death	4
**P45**	73	male	Right-sided	T2 N2b	III	death	12
**P46**	42	female	Right-sided	T3 N0	I	death	0
**P47**	65	female	Left-sided	T3 N1a	III	death	16
**P48**	50	female	Right-sided	T2 N0	I	death	8
**P49**	75	male	Right-sided	T2 N1a	III	death	8
**P50**	61	female	Left-sided	T2 N0	I	survival	68

Table S2 Associations of NK-1R expression with clinicopathological characteristics of 50 patients with colon cancer.

Variables	Total patients (n = 50)	NK-1R expression[a] Negative NK-1R (%)	Positive NK-1R (%)	P [b]
Sex				0.571
Female	26 (52.0)	14 (53.8)	12 (46.2)	
Male	24 (48.0)	11 (45.8)	13 (54.2)	
Age, years				0.544
Median, range	63.6, 38-78	63.1, 46-78	64.0, 38-77	
<60	16 (32.0)	7 (43.8)	9 (56.2)	
≥60	34 (68.0)	18 (52.9)	16 (47.1)	
Tumor site [c]				0.024
Left-sided	24 (48.0)	16 (66.7)	8 (33.3)	
Right-sided	26 (52.0)	9 (34.6)	17 (65.4)	
Tumor grade				0.023
Well	28 (56.0)	18 (64.3)	10 (35.7)	
Poor	22 (44.0)	7 (31.8)	15 (68.2)	
TNM stage [d]				0.004
I + II	30 (60.0)	20 (66.7)	10 (33.3)	
III + IV	20 (40.0)	5 (25.0)	15 (75.0)	

Abbreviation: NK-1R, neurokinin-1 receptor

[a] NK-1R expression was assayed by immunohistochemistry staining

[b] Chi-square test.

[c] The left side of the colon was defined as splenic flexure to rectum, and the other was right-sieded.

[d] The 7th TNM Classification of Malignant Tumors proposed by the AJCC/UICC
